# Chinese Herbal Medicine for Cervicogenic Dizziness: A Systematic Review and Meta-Analysis

**DOI:** 10.1155/2022/2425851

**Published:** 2022-05-09

**Authors:** Hyunjoo Oh, Seungwon Shin, Euiju Lee, Won-Seok Chung

**Affiliations:** ^1^Department of Clinical Korean Medicine, Graduate School, Kyung Hee University, Seoul, Republic of Korea; ^2^National Agency for Development of Innovative Technologies in Korean Medicine, National Development Institute of Korean Medicine, Seoul, Republic of Korea; ^3^Department of Sasang Constitutional Medicine, College of Korean Medicine, Kyung Hee University, Seoul, Republic of Korea; ^4^Department of Rehabilitation Medicine of Korean Medicine, College of Korean Medicine, Kyung Hee University, Seoul, Republic of Korea

## Abstract

**Background:**

Chinese herbal medicines (CHMs) have been widely used in the treatment of cervicogenic dizziness (CGD) based on their empirical effectiveness and safety. Herein, we reviewed and evaluated the clinical evidence of the efficacy and safety of CHMs for CGD.

**Methods:**

Among the relevant studies published in 11 electronic databases up to December 2021, only randomised controlled trials were included. Methodological quality was assessed using the revised Cochrane risk-of-bias tool for randomised trials, and the strength of evidence for the main outcomes was evaluated using the grading of recommendations assessment, development, and evaluation system.

**Results:**

All 35 included randomised controlled trials with 3,862 participants were conducted with six types of modified CHM and four types of active controls. More than half of the included studies were of low quality because of the high risk of bias due to deviations from intended interventions. CHM plus active control was more effective in the treatment of CGD than active control alone. CHM plus anti-vertigo drugs, CHM plus manual therapy, CHM plus acupuncture therapy, and CHM plus manual and acupuncture therapy were all effective in treating CGD, with CHM plus manual and acupuncture therapy showing the most reliable effect. All CHMs were effective for specific patterns of CGD when administered with active controls, with Dingxuan Tang and Yiqi Congming Tang demonstrating the most reliable effects. No serious adverse events were reported in any of the included studies.

**Conclusion:**

The current evidence suggests that CHM may enhance the treatment of CGD when combined with other treatments without serious adverse events. Further high-quality evidence is needed to draw definitive conclusions.

## 1. Introduction

Cervicogenic dizziness (CGD), a major cause of dizziness, is associated with a variety of symptoms, such as headache, unsteadiness, light-headedness, perception of spinning, nausea, and general disorientation, coexisting with neck pain or stiffness [1–4]. Its prevalence is estimated to be 6.4–8.5% [5–7]; however, CGD is common in older patients, especially those with cervical spine dysfunction. Therefore, there is growing apprehension that the number of patients with CGD will increase in accordance with a worldwide ageing population [8–10].

Although it is known that CGD originates from the cervical spine, its pathogenesis remains unclear [11]. Until now, the most prevalent hypothesis is that CGD is caused by disharmonic hyperactivity of the cervical mechanoreceptors located in the joints, ligaments, and muscle spindles, which occurs when the proprioceptive system of the neck is damaged due to muscular fatigue, degeneration, or trauma [10, 12–14]. In a recent review, CGD was classified according to the aetiopathological mechanisms into neural types, comprising degenerative cervical spine disorder, whiplash-associated disorder, and Barré–Liéou syndrome, and vascular types, comprising Bow Hunter's syndrome and Beauty Parlour syndrome. However, these diseases also overlap because they do not have completely distinct mechanisms [15]. Because there are no established diagnostic criteria for CGD, physicians usually diagnose CGD when the patients' symptoms are not related to other neurological or neuro-otological causes of dizziness [16, 17].

The treatment of CGD has not yet been standardised. Previous studies have explored a variety of treatments to improve the severity and frequency of dizziness by relaxing muscles and ameliorating abnormal proprioceptive sensitivity or impaired blood flow in the cervical region. Treatment strategies include physical therapy [1, 3, 7, 10, 18–22], surgery [10, 16], topical drug injection [9, 23], acupuncture therapy [24, 25], and medications, such as muscle relaxants, opioids, nonsteroidal anti-inflammatory drugs, and anxiolytics, in combination with Chinese herbal medicines (CHMs). CHMs have been widely used for CGD, either alone or in combination with other treatments, based on their empirical effectiveness to suppress pain and improve blood circulation in the human body [24, 26]. However, there has been no systematic verification of their efficacy and safety in the treatment of CGD based on clinical evidence.

Therefore, we aimed to review and evaluate the clinical evidence on the efficacy and safety of CHM as monotherapy or adjunctive therapy for CGD, which would promote evidence-based decision-making in clinical practice.

## 2. Methods

### 2.1. Study Registration

The study protocol for this systematic review was registered with the International Prospective Register of Systematic Reviews (registration number: CRD42020199222; registration date: October 27, 2020) and the Research Registry (Review Registry Unique Identifying Number: reviewregistry1036; registration date: November 19, 2020). The study protocol was published [27], and there have been no subsequent amendments that could result in a significant change in the study design. This systematic review is reported in accordance with the Preferred Reporting Items for Systematic Review and Meta-Analyses statement [28]. A preprint has previously been published in Research Square (DOI: https://doi.org/10.21203/rs.3.rs-364098/v1; registration date: March 31, 2021) [29].

### 2.2. Data Sources and Search Strategy

One researcher (HO) comprehensively searched the following 11 electronic databases for relevant studies published up to December 2021 without language or publication status restrictions: three English databases (Medical Literature Analysis and Retrieval System Online (MEDLINE) via PubMed, Excerpta Medica Database (EMBASE) via Elsevier, and the Cochrane Central Register of Controlled Trials (CENTRAL)), six Korean databases (KoreaMed, Korean Studies Information Service System, Research Information Sharing Service, National Digital Science Library, Korean Medical Database, and Database Periodical Information Academic), one Chinese database (China National Knowledge Infrastructure), and one Japanese database (Citation Information by NII). A manual search on Google Scholar was also performed to identify additional eligible studies among those listed in the reference sections of included studies. The search strategies were tailored to the language and search systems of the databases. The search strategies used in the three English databases (MEDLINE, EMBASE, and CENTRAL) are presented in Additional file 1.

### 2.3. Eligibility Criteria

#### 2.3.1. Types of Studies

All randomised controlled trials (RCTs) related to the use of CHMs for CGD were included. All other study designs, including quasi-RCTs, were excluded.

#### 2.3.2. Participants

All patients with CGD were included as subjects in this study, with no restrictions on ethnicity, nationality, sex, age, or biological status.

#### 2.3.3. Interventions and Comparisons

CHMs with any formulation administered orally, such as decoction, capsules, tablets, pills, and powders, were considered experimental interventions. There was no limitation on the number or combination of herbs, CHM dose, or the frequency or duration of treatment. If the composition of CHMs used in the included studies differed from the original prescription, “modified” was indicated in front of the CHM name. No treatment and placebo were considered as control interventions to determine the efficacy of CHM as monotherapy. Active controls, such as anti-vertigo drugs, manual therapy, and acupuncture therapy, were also considered as control interventions to determine the efficacy of CHM as adjunctive therapy only when CHMs were equally applied to both the experimental and control groups. Studies comparing different combinations of CHMs or CHM alone with other active controls were excluded because they could not rigorously determine the efficacy of CHMs.

#### 2.3.4. Outcomes

The primary outcomes were as follows:

1. The change in the patients' overall functional score measured by validated scales (e.g., functional scale for cervical spondylosis of vertebral artery type)
2. The change in the patients' simple score for dizziness (e.g., the numerical rating scale)
3. The change in mean blood flow velocity in the vertebrobasilar artery, as evaluated using transcranial Doppler

The secondary outcomes were as follows:

1. The total effective rate, strictly calculated by counting only the number of patients completely cured, to exclude researcher subjectivity and improve the reliability of the results
2. The changes in haematological parameters, such as fibrinogen levels, endothelin, total cholesterol (TC), and calcitonin gene-related peptide (CGRP)
3. Adverse events

### 2.4. Study Selection Process

Two reviewers (HO and SS) independently screened and assessed all retrieved studies for eligibility based on the aforementioned criteria. After duplicates were removed, the titles and abstracts of the remaining studies were screened using EndNote X9 (Clarivate Analytics, London, UK). Next, the full-text review of the eligible studies was conducted for final inclusion. Any divergence in the agreement was resolved through discussion with a third researcher (EL) at each step of the study selection process.

### 2.5. Data Extraction

Two reviewers independently extracted data from the included studies (HO and SS) using a predefined data acquisition form. This form included four main domains: general information (title, authors, year of publication, country of the study, and study design), participants' characteristics (age, sex, diagnostic criteria, and CGD duration), intervention and comparison details (sample size; CHM formulation and prescription name; number of herbs; CHM dose; CHM daily dose; comparison, frequency, or duration of the treatment; and follow-up information), and outcomes (primary and secondary outcomes and adverse events). Any discrepancies were resolved through discussion with a third researcher (EL).

### 2.6. Quality Assessment

The methodological quality of the included studies was assessed using the revised Cochrane risk-of-bias tool for randomised trials [30]. The bias domain for risk-of-bias assessment included the following: (1) bias arising from the randomisation process, (2) bias due to deviations from intended interventions, (3) bias due to missing outcome data, (4) bias in the measurement of the outcome, and (5) bias in the selection of the reported result. The risk of bias was independently evaluated by two reviewers (HO and SS) as “low,” “high,” or “some concerns.” Any divergence in the agreement was resolved through discussion with other reviewers (EL and WSC). Studies evaluated as “low-risk” in all domains were defined as high-quality studies, whereas those evaluated as “high-risk” in at least one domain were defined as low-quality studies.

Subsequently, the strength of evidence for the main outcomes was evaluated using the grading of recommendations assessment, development, and evaluation system [31]. The risk of bias; inconsistency, indirectness, and imprecision of the results; and publication bias were assessed, and the quality of the evidence was graded on a four-point scale as “high,” “moderate,” “low,” or “very low.”

### 2.7. Data Synthesis

When the included studies were sufficiently homogenous, quantitative synthesis was performed using RevMan software (version 5.3; Cochrane, London, UK) to analyse the efficacy of CHMs in the treatment of CGD. Subgroup analyses were conducted according to (1) the comparison types and (2) the CHM prescription names. Dichotomous outcomes were pooled using risk ratios (RRs), and continuous outcomes were pooled using mean differences (MDs), or standardised mean differences (SMDs), with 95% confidence intervals (CIs).

The statistical heterogeneity among studies was assessed by computing *I*^2^ statistics. Data were pooled using a random-effects model, if the included studies had significant heterogeneity (*I*^2^ values ≥50% indicated substantial heterogeneity and *I*^2^ values ≥75% indicated considerable heterogeneity (both were considered significant)). Otherwise, a fixed-effects model was applied [32]. Sensitivity analysis was performed to increase the robustness of the results by excluding studies with a high risk of bias and outliers. If the number of studies was sufficient (*n* ≥ 10), a visual inspection of the funnel plot was performed to assess publication bias. Data on the safety of CHMs in the treatment of CGD were described qualitatively.

## 3. Results

### 3.1. Study Selection

**Figure 1:**
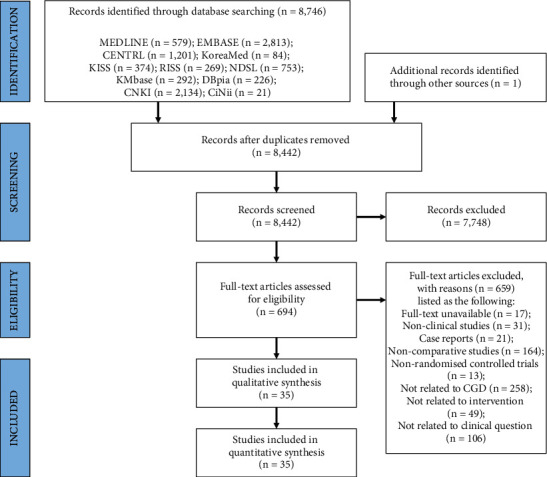
PRISMA flow diagram of the literature screening and selection process. CENTRAL, Cochrane Central Register of Controlled Trials; CGD, cervicogenic dizziness; CiNii, Citation Information by NII; CNKI, China National Knowledge Infrastructure; DBpia, Database Periodical Information Academic; EMBASE, Excerpta Medica Database; KISS, Korean Studies Information Service System; KMbase, Korean Medical Database; MEDLINE, Medical Literature Analysis and Retrieval System Online; NDSL, National Digital Science Library; PRISMA, Preferred Reporting Items for Systematic reviews and Meta-Analyses; and RISS, Research Information Sharing Service.

A total of 8,746 studies were identified through the database searches, and 1 additional study was identified through other sources. After removing 305 duplicates, 8,442 studies were excluded by screening the titles and abstracts. Through a review of the full texts, a further 659 studies were excluded: 17 studies with unavailable full texts, 31 nonclinical studies, 21 case reports, 164 noncomparative studies, 13 nonrandomised controlled trials, 258 studies not related to CGD, 49 studies not related to eligible intervention, and 106 studies not related to the clinical question. Finally, 35 RCTs with 3,862 participants were included in the analysis (Figure 1).

### 3.2. Study Characteristics

All included studies were RCTs conducted in China. They were classified according to the comparison types, as follows: (1) studies comparing CHMs plus anti-vertigo drugs with anti-vertigo drugs alone (*n* = 14), which were subdivided according to the anti-vertigo drugs used into studies using flunarizine (*n* = 6), betahistine (*n* = 5), both flunarizine and betahistine (*n* = 1), diphenidol (*n* = 1), or nimodipine (*n* = 1); (2) studies comparing CHMs plus manual therapy with manual therapy alone (*n* = 7); (3) studies comparing CHMs plus acupuncture therapy with acupuncture therapy alone (*n* = 13); and (4) studies comparing CHMs plus manual and acupuncture therapy with manual and acupuncture therapy alone (*n* = 1). None of the studies assessed the efficacy of CHM as monotherapy for CGD.

The included studies were also classified according to the CHM prescription names, as follows: (1) studies on Banxia Baizhu Tianma Tang (BBTT; *n* = 9), (2) studies on Buzhong Yiqi Tang (BYT; *n* = 2), (3) studies on Dingxuan Tang (DXT; *n* = 8), (4) studies on Gegen Tang (GGT; *n* = 7), (5) study on Gegen Jieji Tang (GJT; *n* = 1), and (6) studies on Yiqi Congming Tang (YCT; *n* = 8). All CHMs in the included studies were modified prescriptions. In summary, the studies included in this review were conducted with six types of modified CHMs (BBTT, BYT, DXT, GGT, GJT, and YCT) and four types of active controls (anti-vertigo drugs, manual therapy, acupuncture therapy, and manual and acupuncture therapy).

In addition, 10 types of outcome measurements were identified: 5 studies evaluated overall functional scores, 22 studies evaluated simple scores, 17 studies assessed the mean blood flow velocity in the vertebral arteries, 18 studies assessed the mean blood flow velocity in the basilar artery, 33 studies evaluated the total effective rate, three studies measured endothelin levels, and four studies measured CGRP, fibrinogen and TC levels. The incidence of adverse events was reported in three studies. The study characteristics and the main outcomes are summarised in Table 1.

Each CHM prescription was applied to a specific pattern of symptoms in traditional Chinese medicine: BBTT to the wind-phlegm type or phlegm stasis type; BYT to qi and blood deficiency type; DXT to spleen deficiency and dampness type, qi deficiency and blood stasis type, or hyperactivity of liver yang type; GGT to wind type with disharmony between ying and wei; GJT to collateral stasis type; and YCT to qi and blood deficiency type or qi deficiency and sputum silting up type. All modified CHMs included at least one-third of the original prescriptions. The duration of administration ranged from 10 days to 8 weeks, with 2- and 4-week regimens being the most frequent. The details of the CHMs prescribed in the included studies are summarised in Tables 2 and 3.

### 3.3. Risk-of-Bias Assessment

**Figure 2:**
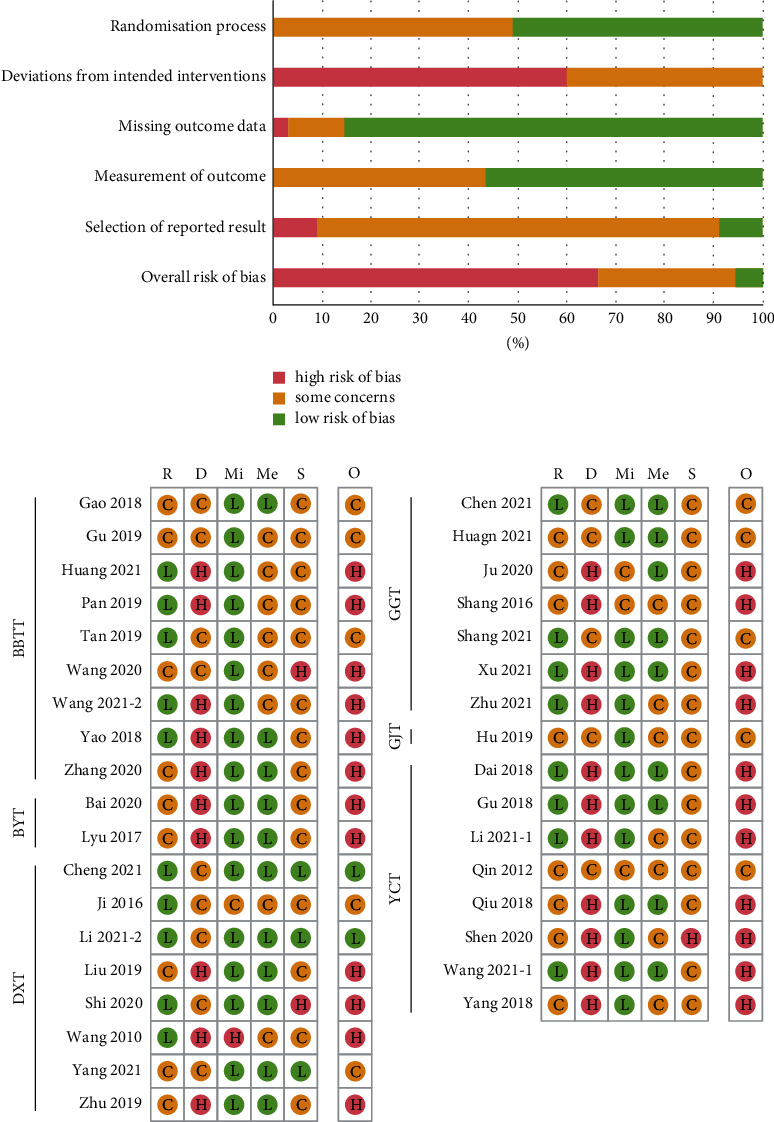
Risk of bias summary for all included studies.

For bias arising from the randomisation process, 18 studies were evaluated as “low-risk” because the randomisation process for the allocation sequence was clearly described. The remaining 17 studies were evaluated as “some concerns” because insufficient relevant information was provided. For bias due to deviations from intended interventions, 21 studies, most of which included manual or acupuncture therapy as active controls, were evaluated as “high-risk” because it was unclear whether blinding of participants and trial personnel had been sufficiently performed using sham-massage or sham-acupuncture. The remaining 14 studies were evaluated as “some concerns.” For bias due to missing outcome data, 30 studies were evaluated as “low-risk,” and 1 study was evaluated as “high-risk” because there were missing data (only the results of the per-protocol analysis were reported). The remaining 4 studies were evaluated as “some concerns” because insufficient relevant information was provided. For bias in the measurement of the outcome, 20 studies were evaluated as “low-risk,” and the remaining 15 studies were evaluated as “some concerns” because it was difficult to judge whether the outcome measures used in the studies were affected by the awareness of the outcome assessors. For bias in the selection of the reported result, 3 studies were evaluated as “low-risk” because there was no suspicion of deliberate nonreporting, and 3 studies were evaluated as “high-risk” because selective outcome reporting was suspected. The remaining 29 studies were evaluated as “some concerns” because there was no basis for bias assessment (e.g., study protocols). Finally, for the overall risk of bias, 23 studies assessed as “high-risk” were considered low-quality studies; 2 were considered high-quality studies; and the remaining 10 studies were evaluated as “some concerns” (Figure 2).

The risk of bias was evaluated as “low,” “high,” or “some concerns,” represented by the following symbols: “L,” “H,” and “C,” respectively. *D*, bias due to deviations from intended interventions; *Me*, bias in the measurement of the outcome; *Mi*, bias due to missing outcome data; *O*, overall risk of bias; *R*, bias arising from the randomisation process; and *S*, bias in the selection of the reported result.

### 3.4. Efficacy

In the total analysis of all included studies, compared with the active controls alone, CHMs plus active controls significantly reduced the overall functional scores (five studies: SMD, 2.31 (95% CI: 1.48–3.14); *I*^2^ = 94%), endothelin (three studies: MD, 14.57 (95% CI: 6.81–22.32); *I*^2^ = 96%), fibrinogen (four studies: MD, 0.31 (95% CI: 0.12–0.50); *I*^2^ = 97%), and TC levels (four studies: MD, 0.56 (95% CI: 0.31–0.82); *I*^2^ = 71%). In addition, CHMs plus active controls significantly increased the simple scores (22 studies: SMD, 1.82 (95% CI: 1.26–2.38); *I*^2^ = 97%), the blood flow velocity in the left vertebral artery (17 studies: MD, 5.70 (95% CI: 4.18–7.22); *I*^2^ = 97%), right vertebral artery (17 studies: MD, 4.83 (95% CI: 3.37–6.29); *I*^2^ = 97%), basilar artery (18 studies: MD, 5.58 (95% CI: 4.24–6.92); *I*^2^ = 96%), CGRP levels (four studies: MD, 6.24 (95% CI: 4.37–8.11); *I*^2^ = 96%), and total effective rate (33 studies: RR, 1.55 (95% CI: 1.42–1.69); *I*^2^ = 0%).

#### 3.4.1. CHMs plus Anti-Vertigo Drugs versus Anti-Vertigo Drugs Alone

In the subanalysis of the 14 studies using anti-vertigo drugs as active controls, compared with the anti-vertigo drugs alone, CHMs plus anti-vertigo drugs significantly reduced the overall functional scores (one study: MD, 7.80 (95% CI: 6.02–9.58)) and endothelin levels (one study: MD, 11.14 (95% CI: 9.49–12.79)). In addition, CHMs plus anti-vertigo drugs significantly increased the simple scores (seven studies: SMD, 2.45 (95% CI: 1.32–3.58); *I*^2^ = 98%), the blood flow velocity in the left vertebral artery (seven studies: MD, 5.39 (95% CI: 3.33–7.45); *I*^2^ = 98%), right vertebral artery (seven studies: MD, 5.28 (95% CI: 3.38–7.18); *I*^2^ = 97%), and basilar artery (seven studies: MD, 5.28 (95% CI: 3.97–6.59); *I*^2^ = 92%). CHMs plus anti-vertigo drugs also significantly improved the total effective rate (13 studies: RR, 1.53 (95% CI: 1.35–1.73); *I*^2^ = 21%). However, the changes in the CGRP levels (two studies: MD, 8.89 (95% CI: −0.76–18.54); *I*^2^ = 98%) did not show a significant difference between the intervention and control groups.

In the additional subanalysis of the components of anti-vertigo drug, the combination of CHMs and flunarizine significantly increased the simple scores (three studies: SMD, 2.16 (95% CI: 0.44–3.87); *I*^2^ = 97%), the blood flow velocity in the left vertebral artery (two studies: MD, 3.96 (95% CI: 1.91–6.01); *I*^2^ = 94%), right vertebral artery (two studies: MD, 4.80 (95% CI: 4.23–5.38); *I*^2^ = 0%), basilar artery (two studies: MD, 4.85 (95% CI: 4.04–5.65); *I*^2^ = 0%), CGRP levels (one study: MD, 13.89 (95% CI: 11.48–16.30)), and the total effective rate (six studies: RR, 1.48 (95% CI: 1.16–1.90); *I*^2^ = 50%). The combination of CHMs and betahistine significantly reduced the overall functional scores (one study: MD, 7.80 (95% CI: 6.02–9.58)) and increased the blood flow velocity in the left vertebral artery (two studies: MD, 8.73 (95% CI: 5.49–11.97); *I*^2^ = 94%), right vertebral artery (two studies: MD, 7.77 (95% CI: 7.17–8.37); *I*^2^ = 25%), basilar artery (two studies: MD, 5.70 (95% CI: 5.15–6.24); *I*^2^ = 0%), and the total effective rate (four studies: RR, 1.68 (95% CI: 1.27–2.23); *I*^2^ = 0%). However, the changes in the simple scores (two studies: SMD, 1.29 (95% CI: −0.34–2.91); *I*^2^ = 98%) did not show a significant difference between the intervention and control groups. The combination of CHMs with flunarizine and betahistine significantly increased the simple scores (one study: MD, 6.98 (95% CI: 6.48–7.48)), the blood flow velocity in the left vertebral artery (one study: MD, 4.59 (95% CI: 3.28–5.90)), right vertebral artery (one study: MD, 5.04 (95% CI: 3.85–6.23)), basilar artery (one study: MD, 6.92 (95% CI: 5.74–8.10)), and the total effective rate (one study: RR, 1.97 (95% CI: 1.29–3.00)). The combination of CHMs and diphenidol significantly increased the simple scores (one study: MD, 2.67 (95% CI: 2.41–2.93)), the blood flow velocity in the left vertebral artery (one study: MD, 5.51 (95% CI: 4.39–6.63)), right vertebral artery (one study: MD, 4.69 (95% CI: 3.77–5.61)), basilar artery (one study: MD, 6.23 (95% CI: 4.42–8.04)), CGRP levels (one study: MD, 4.04 (95% CI: 3.68–4.40)), and reduced endothelin levels (one study: MD, 11.14 (95% CI: 9.49–12.79)). However, the changes in the total effective rate (one study: RR, 1.40 (95% CI: 0.80–2.44)) did not show a significant difference between the intervention and control groups. The combination of CHMs and nimodipine significantly increased the blood flow velocity in the left vertebral artery (one study: MD, 2.40 (95% CI: 1.90–2.90)), right vertebral artery (one study: MD, 1.82 (95% CI: 1.35–2.29)), and basilar artery (one study: MD, 2.74 (95% CI: 2.19–3.29)). However, the changes in the total effective rate (one study: RR, 1.32 (95% CI: 0.85–2.04)) did not show a significant difference between the intervention and control groups.

#### 3.4.2. CHMs plus Manual Therapy versus Manual Therapy Alone

In the subanalysis of the seven studies using manual therapy as an active control, compared with the manual therapy alone, CHMs plus manual therapy significantly increased the simple scores (seven studies: SMD, 1.33 (95% CI: 0.12–2.54); *I*^2^ = 98%), the blood flow velocity in the left vertebral artery (three studies: MD, 6.24 (95% CI: 1.36–11.12); *I*^2^ = 98%), right vertebral artery (three studies: MD, 5.62 (95% CI: 1.03–10.21); *I*^2^ = 98%), basilar artery (three studies: MD, 4.62 (95% CI: 0.32–8.91); I^2^ = 97%), and CGRP levels (two studies: MD, 4.63 (95% CI: 2.25–7.00); *I*^2^ = 93%). Furthermore, CHMs plus manual therapy significantly improved the total effective rate (six studies: RR, 1.71 (95% CI: 1.36–2.16); *I*^2^ = 0%). However, the changes in the overall functional scores (two studies: SMD, 3.17 (95% CI: −0.15–6.48); *I*^2^ = 98%) and endothelin levels (two studies: MD, 16.48 (95% CI: −0.34–33.31); *I*^2^ = 98%) did not show significant differences between the intervention and control groups.

#### 3.4.3. CHMs plus Acupuncture Therapy versus Acupuncture Therapy Alone

In the subanalysis of the thirteen studies using acupuncture therapy as an active control, compared with the acupuncture therapy alone, CHMs plus acupuncture therapy significantly reduced the overall functional scores (one study: MD, 1.91 (95% CI: 1.37–2.45)), fibrinogen (four studies: MD, 0.31 (95% CI: 0.12–0.50); *I*^2^ = 97%), and TC levels (four studies: MD, 0.56 (95% CI: 0.31–0.82); *I*^2^ = 71%). In addition, CHMs plus acupuncture therapy significantly increased the simple scores (eight studies: SMD, 1.72 (95% CI: 1.33–2.11); *I*^2^ = 79%), the blood flow velocity in the left vertebral artery (seven studies: MD, 5.81 (95% CI: 2.92–8.70); *I*^2^ = 95%), right vertebral artery (seven studies: MD, 4.03 (95% CI: 1.05–7.01); *I*^2^ = 96%), basilar artery (eight studies: MD, 6.43 (95% CI: 2.97–9.89); *I*^2^ = 97%), and the total effective rate (thirteen studies: RR, 1.54 (95% CI: 1.32–1.78); *I*^2^ = 0%).

#### 3.4.4. CHMs plus Manual and Acupuncture Therapy versus Manual and Acupuncture Therapy Alone

In the subanalysis of the one study using manual and acupuncture therapy as an active control, CHMs plus manual and acupuncture therapy significantly reduced the overall functional scores (one study: MD, 7.06 (95% CI: 6.27–7.85)) and improved the total effective rate (one study: RR, 1.40 (95% CI: 1.02–1.94)), compared with the active control alone.

#### 3.4.5. BBTT plus Active Controls versus Active Controls Alone

In the subanalysis of the nine studies using BBTT as CHM, compared with the active controls alone, BBTT plus active controls significantly reduced the overall functional scores (two studies: SMD, 3.44 (95% CI: 0.69–6.20); *I*^2^ = 98%) and endothelin levels (one study: MD, 25.13 (95% CI: 21.29–28.97)) and increased the simple scores (two studies: MD, 5.15 (95% CI: 4.81–5.50); *I*^2^ = 0%), the blood flow velocity in the left vertebral artery (two studies: MD, 4.44 (95% CI: 3.18–5.69); *I*^2^ = 71%), right vertebral artery (two studies: MD, 3.85 (95% CI: 2.29–5.41); *I*^2^ = 84%), basilar artery (two studies: MD, 3.48 (95% CI: 0.04–6.92); *I*^2^ = 95%), and CGRP levels (one study: MD, 5.89 (95% CI: 4.78–7.00)). BBTT plus active controls also significantly improved the total effective rate (nine studies: RR, 1.48 (95% CI: 1.29–1.70); *I*^2^ = 33%).

#### 3.4.6. BYT plus Active Controls versus Active Controls Alone

In the subanalysis of the two studies using BYT as CHM, compared with the acupuncture therapy alone, BYT plus acupuncture therapy significantly increased the simple scores (two studies: MD, 2.04 (95% CI: 1.35–2.72); *I*^2^ = 0%) and the blood flow velocity in the left vertebral artery (two studies: MD, 1.72 (95% CI: 0.57–2.87); *I*^2^ = 0%). However, the changes in the blood flow velocity in the basilar artery (two studies: MD, 0.43 (95% CI: −0.68–1.55); *I*^2^ = 0%) and the total effective rate (two studies: RR, 1.27 (95% CI: 0.70–2.28); *I*^2^ = 0%) did not show significant differences between the intervention and control groups. Notably, the blood flow velocity in the right vertebral artery (two studies: MD, −1.80 (95% CI: −2.88–0.72); *I*^2^ = 0%) showed a significant increase in the control group compared with the intervention group.

#### 3.4.7. DXT plus Active Controls versus Active Controls Alone

In the subanalysis of the eight studies using DXT as CHM, compared with the active controls alone, DXT plus active controls significantly reduced the overall functional scores (one study: MD, 5.68 (95% CI: 4.36–7.00)) and endothelin levels (two studies: MD, 9.71 (95% CI: 6.61–12.81); *I*^2^ = 76%) and increased the simple scores (seven studies: SMD, 1.67 (95% CI: 0.20–3.14); *I*^2^ = 98%), the blood flow velocity in the left vertebral artery (five studies: MD, 5.13 (95% CI: 3.87–6.40); *I*^2^ = 78%), right vertebral artery (five studies: MD, 5.12 (95% CI: 3.42–6.83); *I*^2^ = 90%), basilar artery (five studies: MD, 5.14 (95% CI: 2.66–7.62); *I*^2^ = 92%), and CGRP levels (three studies: MD, 6.41 (95% CI: 4.15–8.67); *I*^2^ = 97%). Moreover, DXT plus active controls significantly improved the total effective rate (eight studies: RR, 1.61 (95% CI: 1.33–1.95); *I*^2^ = 0%).

#### 3.4.8. GGT plus Active Controls versus Active Controls Alone

In the subanalysis of the seven studies using GGT as CHM, compared with the active controls alone, GGT plus manual therapy significantly reduced the overall functional scores (one study: MD, 7.80 (95% CI: 6.02–9.58)) and increased the simple scores (five studies: SMD, 1.92 (95% CI: 0.99–2.85); *I*^2^ = 94%), the blood flow velocity in the left vertebral artery (five studies: MD, 7.29 (95% CI: 3.51–11.07); *I*^2^ = 99%), right vertebral artery (five studies: MD, 6.18 (95% CI: 3.12–9.24); *I*^2^ = 99%), and basilar artery (five studies: MD, 5.19 (95% CI: 3.50–6.88); *I*^2^ = 96%). Moreover, GGT plus active controls significantly improved the total effective rate (six studies: RR, 1.62 (95% CI: 1.32–1.99); *I*^2^ = 0%).

#### 3.4.9. GJT plus Active Controls versus Active Controls Alone

In the subanalysis of the one study using GJT as CHM, compared with the betahistine alone, GJT plus betahistine significantly increased the simple scores (one study: MD, 2.00 (95% CI: 1.75–2.25)). However, the total effective rate (one study: RR, 2.19 (95% CI: 0.99–4.86)) was not significantly different between the intervention and control groups.

#### 3.4.10. YCT plus Active Controls versus Active Controls Alone

In the subanalysis of the eight studies using YCT as CHM, compared with the active controls alone, YCT plus active controls significantly reduced the overall functional scores (one study: MD, 1.91 (95% CI: 1.37–2.45)), fibrinogen (four studies: MD, 0.31 (95% CI: 0.12–0.50); *I*^2^ = 97%) and TC levels (four studies: MD, 0.56 (95% CI: 0.31–0.82); *I*^2^ = 71%) and increased the simple scores (five studies: SMD, 1.79 (95% CI: 0.93–2.64); *I*^2^ = 94%), blood flow velocity in the left vertebral artery (three studies: MD, 7.63 (95% CI: 4.69–10.57); *I*^2^ = 80%), right vertebral artery (three studies: MD, 7.34 (95% CI: 6.02–8.66); *I*^2^ = 0%), and basilar artery (four studies: MD, 11.01 (95% CI: 4.46–17.56); *I*^2^ = 96%). Furthermore, YCT plus active controls significantly improved the total effective rate (seven studies: RR, 1.54 (95% CI: 1.28–1.84); *I*^2^ = 0%).

Summarizing the results of the subanalysis according to CHM prescription names, BBTT, DXT, GGT, and YCT showed significant treatment effects on various primary and secondary outcomes and had relatively more clinical evidence compared with the remaining CHM prescription names. GJT was investigated in only one RCT and demonstrated a significant effect on only one primary outcome (change in the simple scores), without statistically significant effects on the other outcome (total effective rate). In the two RCTs investigating BYT, there were significant effects on two primary outcomes (change in the simple scores and blood flow velocity in the left vertebral artery), while the effects on the remaining outcomes were either not significant (blow flow velocity in the basilar artery and total effective rate) or were significant in the control group (the blood velocity for the right vertebral artery). The results of the total analysis and the subanalyses of the efficacy of CHMs are shown in Table 4.

### 3.5. Safety

Three of the thirty-five included studies reported adverse events. There was one case of gastrointestinal discomfort in the BBTT plus manual therapy group; one case of abdominal pain; one case of fainting during acupuncture therapy in the BBTT plus acupuncture therapy group; one case of rash, diarrhea, and gastrointestinal discomfort each, and two cases of fatigue in the DXT plus anti-vertigo drugs (flunarizine and betahistine) group. All reported adverse events were mild and transient and were evaluated as “not serious” (Table 1).

### 3.6. Quality of Evidence

In the comparison of CHMs plus active controls versus active controls alone, the quality of evidence for the primary outcomes ranged from “very low” to “low.” For the secondary outcomes, the quality of evidence for the total effective rate was graded as “moderate,” while that for the other outcomes was graded as “very low” or “low.” The overall quality of evidence in the total analysis was graded as “low.” In the subanalysis based on the type of active control, the overall quality of evidence was graded as “moderate” for CHMs plus manual and acupuncture therapy and as “low” for CHMs plus any other active control (anti-vertigo drugs, manual therapy, or acupuncture therapy). In the subanalysis based on the CHM prescription name, the overall quality of evidence was evaluated as “low” for all CHM prescriptions. However, its quantitative and qualitative levels were highest for DXT and YCT and lowest for BYT and GJT, respectively. The main reason for the downgrade was the high risk of bias in the included studies, the imprecision of the results due to the small sample size, and the inconsistency of the results due to the high heterogeneity among them (Table 4).

### 3.7. Sensitivity Analysis

For the outcomes with considerable heterogeneity among studies, we performed sensitivity analysis and adjusted the quality of evidence based on the results. After heterogeneity was eliminated by removing one to two outliers considered to have a high risk for selection and reporting biases, the quality of evidence for the efficacy of CHMs for CGD was similar to that obtained before the sensitivity analysis. Therefore, the findings in this systematic review are considered robust to the decisions made in the process of obtaining them (Table 5).

### 3.8. Publication Bias

**Figure 3:**
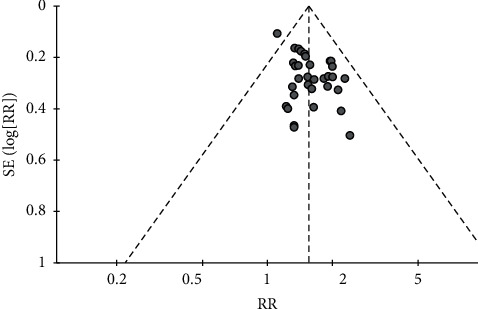
Funnel plot of the effects of CHMs plus active controls on the total effective rate.

**Figure 4:**
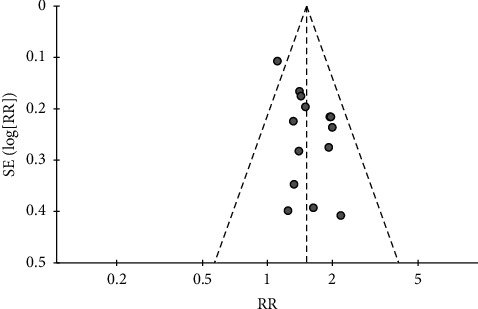
Funnel plot of the effects of CHMs plus anti-vertigo drugs on the total effective rate.

**Figure 5:**
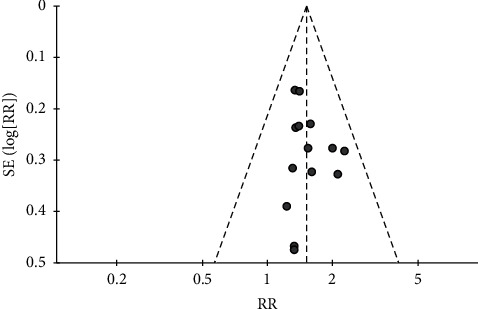
Funnel plot of the effects of CHMs plus acupuncture therapy on the total effective rate.

**Figure 6:**
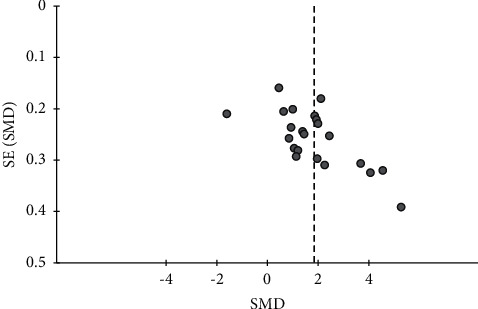
Funnel plot of the effects of CHMs plus active controls on the simple scores.

**Figure 7:**
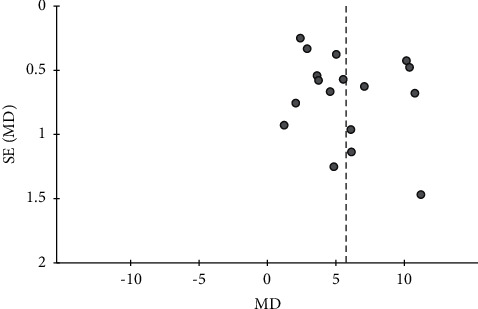
Funnel plot of the effects of CHMs plus active controls on the blood flow velocity in the left vertebral artery.

**Figure 8:**
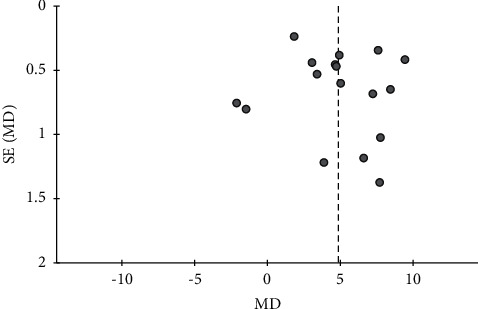
Funnel plot of the effects of CHMs plus active controls on the blood flow velocity in the right vertebral artery.

**Figure 9:**
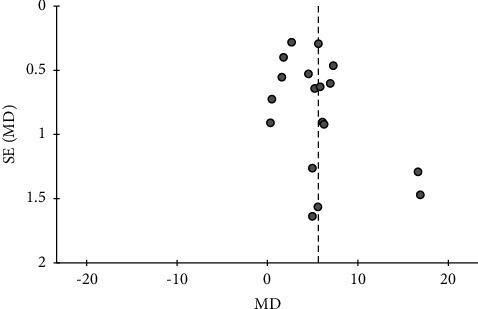
Funnel plot of the effects of CHMs plus active controls on the blood flow velocity in the basilar artery.

For seven outcomes included in more than ten studies, we examined publication bias using funnel plot analysis. For the comparisons of CHMs plus active controls, anti-vertigo drugs, or acupuncture therapy versus active controls, anti-vertigo drugs, or acupuncture therapy alone, respectively, the funnel plots of the total effective rate were symmetrical for all (Figures 3–5). Conversely, for the comparison of CHMs plus active controls versus active controls, the funnel plots of the simple scores and the blood flow velocity in the vertebrobasilar arteries showed asymmetry. In the funnel plot of the blood flow velocity in the left vertebral artery, the asymmetry was presumed to be due to considerable heterogeneity. The asymmetry for the remaining outcomes suggested potential publication bias; thus, there may be negative results not published in the literature (Figures 6–9).

## 4. Discussion

### 4.1. Summary of Findings

In this study, we reviewed and evaluated the available clinical evidence on the efficacy and safety of CHM as monotherapy or adjunctive therapy in the treatment of CGD to promote evidence-based decision-making in clinical practice. As none of the 35 included RCTs [33–67] assessed the efficacy of CHM as monotherapy for CGD, we evaluated its efficacy as adjunctive therapy in combination with other active controls. The included studies were conducted with 6 types of modified CHMs and 4 types of active controls. In the risk-of-bias assessment, more than half of the included studies were considered to be of low quality because of the high risk of bias due to deviations from intended interventions. The results of the efficacy analyses of CHMs plus active controls indicated the following. First, CHMs plus active controls were more effective in treating CGD than active controls alone (the duration of administration ranged from 10 days to 8 weeks). Second, CHMs plus anti-vertigo drugs (flunarizine/betahistine/flunarizine and betahistine/diphenidol/nimodipine), CHMs plus manual therapy, CHMs plus acupuncture therapy, and CHMs plus manual and acupuncture therapy were all effective in treating CGD. Among all, CHMs plus manual and acupuncture therapy showed the most reliable effect. Third, BBTT, BYT, DXT, GGT, GJT, and YCT were effective for specific patterns in patients with CGD, when administered with active controls. Among the CHM prescriptions, DXT and YCT exhibited the most reliable effects, when combined with active controls. Regarding the safety of CHMs plus active controls in the treatment of CGD, no serious adverse events were reported in any of the included studies.

### 4.2. Implications for Clinical Practice

In traditional Chinese medicine, CHMs are prescribed to match the specific pattern of the patients' signs and symptoms. It is reasonable to select and prescribe the most appropriate CHM for a specific pattern in each patient with CGD, as opposed to consistently prescribing one CHM to all patients with CGD, even if it is the most evidence-based prescription for CGD. Thus, although DXT and YCT had the highest level of clinical evidence for the treatment effect on CGD in this review, it may be more effective to use other CHMs for specific patterns in some patients with CGD. In traditional Chinese medicine, wind, fire, phlegm, blood stasis, and deficiency are considered the main pathogenetic factors for CGD [25]. DXT is usually prescribed for CGD syndromes of spleen deficiency and dampness, qi deficiency and blood stasis, or hyperactivity of liver yang. DXT has the effect of removing a pathogenic mass as the original prescription, and it can be prescribed for both deficiency and excess syndromes by modification of the original prescription. CHMs can be modified for better efficacy and fewer side effects [68]. In cases of combined excess and deficiency syndromes, such as spleen deficiency and dampness type or qi deficiency and blood stasis type, DXT was modified by the addition of herbs that have effects on invigorating the qi and spleen (*Codonopsis pilosulae* Radix and *Atractylodis* Rhizoma Alba), regulating qi-flowing (Citri Reticulatae Pericarpium), enriching the blood (*Angelicae* Gigantis Radix), and soothing the nerves (Fossilia Ossis Mastodi), but with subtraction of other herbs from the original prescription, which have effects on suppressing hyperactive liver for calming endogenous wind (*Uncariae* Ramulus Cum Uncis and Scorpio) and promoting blood circulation while removing blood stasis (*Salviae miltiorrhizae* Radix) [43, 47]. Conversely, in cases of excess syndrome only, such as hyperactivity of liver yang type, DXT was modified by adding *Puerariae* Radix, which has the effect of dispelling wind-heat [57, 66]. For the combination of DXT and other treatments, quantitative clinical evidence has been reported for the use of DXT with manual therapy [47, 57, 66]. Both YCT and BYT are usually prescribed for CGD syndromes of qi and blood deficiency, while YCT is also used for the sputum silting up type. The clinical evidence for YCT is better than that for BYT because the latter showed low precision for outcomes. For the combination of YCT and other treatments, the majority of quantitative clinical evidence was reported for the use of YCT with acupuncture therapy [36, 38, 45, 51, 54, 59, 62]. BBTT, which has the effect of dispelling pathogenic wind and eliminating phlegm, is usually prescribed for CGD syndromes of wind-phlegm or phlegm stasis [37, 39, 56]. GGT is usually prescribed for CGD syndromes of wind with disharmony between ying and wei [52]. Both BBTT and GGT were used with various active controls and showed reliable treatment effects. GJT was used for the collateral stasis type with betahistine [40]. Through this review, we gain a clue about the relationship between specific patterns of CGD and CHM prescriptions; however, it remains unknown which CHM prescription is most effective for specific patterns of CGD because all included studies used only one CHM prescription with one specific pattern of CGD. Furthermore, studies are needed to confirm which CHM prescription is most effective for specific patterns of CGD.

### 4.3. Implications for Research

In this review, we identified fibrinogen, endothelin, TC, and CGRP as haematological parameters used in clinical studies on CGD. Endothelin and CGRP were used as indicators to determine the efficacy of CHMs plus anti-vertigo drugs and CHMs plus manual therapy. Fibrinogen and TC were used to determine the efficacy of CHMs plus acupuncture therapy. Endothelin is an endogenous vasoconstrictor that reduces the perfusion of brain tissues by constricting the blood vessels in the brain [69, 70]. CGRP is a vasodilator, mainly distributed in the central nervous system [71]. In a previous study, endothelin and CGRP were reported as important factors affecting the development of CGD with vertebrobasilar arteriospasm [72]. Fibrinogen also promotes the formation of artherosclerotic plaques [73], and TC accelerates atherosclerosis and causes lipid metabolism disorders [74]. In summary, control of endothelin and CGRP levels improves the prognosis of patients with CGD, and evaluation of fibrinogen and TC levels helps predict CGD progression. Therefore, it is recommended to use them as outcomes when conducting further clinical trials of CGD.

This research is valuable as the first systematic review to comprehensively evaluate the efficacy and safety of CHMs in treating CGD, to guide clinicians in selecting and prescribing suitable CHMs for specific patterns of CGD based on evidence-based decision-making. Furthermore, it provides knowledge of which treatments will be effective in combination with CHMs. This review may contribute to the development of effective strategies for the treatment and management of an increasing number of patients with CGD due to population ageing. Nonetheless, further high-quality evidence from rigorously conducted clinical studies, preferably conducted outside China, is required to support the clinical recommendations regarding the use of CHMs for CGD. In addition, placebo-controlled RCTs are needed to evaluate the efficacy of CHMs as monotherapy for CGD. Furthermore, experimental studies of the mechanism of action and the dose-response relationship of CHMs are necessary to determine the optimal dose.

## 5. Limitations

This review has some limitations. First, some of the major Chinese databases, such as Wanfang and VIP, were not included in the search process. Additionally, grey literature was not considered. Thus, there is a possibility that relevant studies were omitted. Second, the quality of the included RCTs was generally poor, in particular, because of the high risk of bias due to deviations from intended interventions. Third, most meta-analyses showed high heterogeneity among studies. Fourth, potential publication bias could not be ruled out because the assessment of publication bias was not conducted in the meta-analyses in which the number of included studies was less than 10, and all RCTs were conducted in China and published in Chinese. Fifth, there is the possibility of attrition bias because few studies presented dropout or withdrawal statistics. Sixth, it is unknown whether the treatment effect of CHMs plus active controls was maintained after completion of the intervention because most studies did not perform follow-up assessments. Finally, the safety of CHMs in patients with CGD remains unknown because few studies clearly reported that there were no adverse events.

## 6. Conclusions

Current evidence suggests that CHMs may have the potential to enhance the treatment effect on CGD when combined with other treatments without serious adverse events. As the overall quality of the studies included in this review was generally low, additional high-quality evidence is needed to draw definitive conclusions.

## Figures and Tables

**Table 1:** General characteristics of the included studies.

Study ID	Sample size (A : B)	Study of the country	Mean age (range; yr)	CGD duration (range)	Intervention group (A)	Control group (B)	Treatment duration	Follow-up	Outcome	Results	AE (*n*)
Bai [33]	80 (40 : 40)	China	(A) 35.6 ± 6.4 (22∼54) (B) 36.2 ± 7.2 (22∼55)	(A) NR (1.5 days∼4 yr) (B) 1.0 ± 0.6 yr (2 days∼4 yr)	Modified BYT + (B)	AT (1 time/day)	20 days	NR	(1) SS (2) RVA-BF (3) LVA-BF (4) BA-BF (5) TER	(1) (A) > (B)^*∗*^ (2) N.S. (3) (A) > (B)^*∗*^ (4) (A) > (B)^*∗*^ (5) (A) > (B)^*∗*^	NR
Chen [34]	120 (60 : 60)	China	(A) 43.81 ± 5.57 (25∼58) (B) 43.75 ± 5.61 (22∼57)	(A) 7.65 ± 1.79 mon (2∼11 mon) (B) 7.63 ± 1.82 mon (2∼11 mon)	Modified GGT + (B)	AD: flunarizine (10 mg bid)	1 month	NR	(1) SS (2) RVA-BF (3) LVA-BF (4) BA-BF (5) TER	(1) (A) > (B)^*∗*^ (2) (A) > (B)^*∗*^ (3) (A) > (B)^*∗*^ (4) (A) > (B)^*∗*^ (5) (A) > (B)^*∗*^	NR
Cheng [35]	84 (42 : 42)	China			Modified DXT + (B)	AD: flunarizine (10 mg qd)	2 weeks	NR	(1) SS (2) TER (3) CGRP level	(1) (A) > (B)† (2) (A) > (B)^*∗*^ (3) (A) > (B)†	NR
Dai [36]	82 (41 : 41)	China	46.2 ± 5.1 (24∼65)	NR (3∼11 mon)	Modified YCT + (B)	AT (1 time/day)	4 weeks	NR	(1) TER (2) Fib level (3) TC level	(1) (A) > (B)^*∗*^ (2) (A) > (B)^*∗*^ (3) (A) > (B)^*∗*^	NR
Gao [37]	106 (53 : 53)	China	(A) 54.3 ± 5.6 (24∼62) (B) 55.4 ± 5.2 (23∼63)	(A) 5.4 ± 0.6 yr (3.0∼10.5 yr) (B) 5.6 ± 0.5 yr (3.5∼11.0 yr)	Modified BBTT + (B)	AD: flunarizine (5∼10 mg·qd)	2 weeks	NR	(1) RVA-BF (2) LVA-BF (3) BA-BF (4) TER	(1) (A) > (B)^*∗*^ (2) (A) > (B)^*∗*^ (3) (A) > (B)^*∗*^ (4) (A) > (B)^*∗*^	NR
Gu [38]	70 (35 : 35)	China	52.17 ± 6.34 (24∼65)	3.08 ± 0.41 mon (2∼6 mon)	Modified YCT + (B)	AT (1 time/2 day)	4 weeks	NR	(1) SS (2) OFS (3) RVA-BF (4) LVA-BF (5) BA-BF (6) TER (7) Fib level (8) TC level	(1) (A) > (B)† (2) (A) > (B)† (3) (A) > (B)^*∗*^ (4) (A) > (B)^*∗*^ (5) (A) > (B)^*∗*^ (6) (A) > (B)^*∗*^ (7) (A) > (B)† (8) (A) > (B)†	NR
Gu [39]	80 (40 : 40)	China	(A) 41.9 ± 5.6 (20∼64) (B) 41.3 ± 5.3 (21∼63)	NR	Modified BBTT + (B)	AD: flunarizine (10∼20 mg·qd)	2∼8 weeks	NR	(1) TER	(1) (A) > (B)†	NR
Hu [40]	200 (120 : 80)	China	(A) 55.71 ± 6.93 (B) 56.43 ± 7.34	(A) 10.37 ± 3.23 yr (B) 10.53 ± 4.12 yr	Modified GJT + (B)	AD: betahistine (6 mg tid)	2 weeks	NR	(1) SS (2) TER	(1) (A) > (B)^*∗*^ (2) (A) > (B) †	NR
Huagn [41]	98 (49 : 49)	China	(A) 67.82 ± 5.95 (B) 65.26 ± 5.43	(A) 3.28 ± 0.69 yr (B) 3.34 ± 0.75 yr	Modified GGT + (B)	AD: betahistine (6 mg·tid)	2 weeks	NR	(1) OFS (2) RVA-BF (3) LVA-BF (4) BA-BF (5) TER	(1) (A) > (B)† (2) (A) > (B)† (3) (A) > (B)† (4) (A) > (B)† (5) (A) > (B)^*∗*^	NR
Huang [42]	120 (60 : 60)	China	(A) 43.63 ± 4.72 (25∼57) (B) 43.72 ± 4.54 (27∼58)	(A) 5.70 ± 1.14 yr (4 mon∼10 yr) (B) 5.65 ± 1.21 yr (3 mon∼10 yr)	Modified BBTT + (B)	MT: Tuina (1 time/2 days)	1 month	NR	(1) SS (2) OFS (3) TER	(1) (A) > (B)^*∗*^ (2) (A) > (B)^*∗*^ (3) (A) > (B)^*∗*^	NR
Ji [43]	60 (30 : 30)	China	NR (40∼70)	NR	Modified DXT + (B)	AD: flunarizine (5 mg·qd)	2 weeks	NR	(1) SS (2) TER	(1) (A) > (B)^*∗*^ (2) (A) > (B)^*∗*^	NR
Ju [44]	120 (60 : 60)	China	(A) 67.82 ± 2.41 (60∼75) (B) 67.91 ± 2.37 (64∼74)	(A) 5.12 ± 0.82 yr (1∼9 yr) (B) 5.30 ± 0.85 yr (1∼10 yr)	Modified GGT + (B)	AT (6 times/week)	4 weeks	6 months	(1) SS (2) RVA-BF (3) LVA-BF (4) BA-BF (5) TER	(1) (A) > (B)^*∗*^ (2) (A) > (B)^*∗*^ (3) (A) > (B)^*∗*^ (4) (A) > (B)^*∗*^ (5) (A) > (B)^*∗*^	NR
Li [45]	68 (34 : 34)	China	(A) 52.60 ± 2.58 (25∼68) (B) 42.58 ± 2.65 (24∼65)	(A) 3.95 ± 0.78 mon (2∼8 mon) (B) 3.92 ± 0.85 mon (1∼8 mon)	Modified YCT + (B)	AT (1 time/day)	4 weeks	NR	(1) SS (2) TER	(1) (A) > (B)† (2) (A) > (B)^*∗*^	NR
Li [46]	116 (58 : 58)	China	(A) 42.98 ± 9.21 (33∼63) (B) 42.91 ± 9.45 (32∼62)	(A) 5.37 ± 0.65 yr (1 mon∼10 yr) (B) 5.32 ± 0.61 yr (1 mon∼10 yr)	Modified DXT + (B)	AD: diphenidol (tid)	1 month	NR	(1) SS (2) RVA-BF (3) LVA-BF (4) BA-BF (5) TER (6) ET level (7) CGRP level	(1) (A) > (B)^*∗*^ (2) (A) > (B)^*∗*^ (3) (A) > (B)^*∗*^ (4) (A) > (B)^*∗*^ (5) (A) > (B)^*∗*^ (6) (A) > (B)^*∗*^ (7) (A) > (B)^*∗*^	NR
Liu [47]	126 (63 : 63)	China	(A) 52.64 ± 8.25 (26∼68) (B) 52.47 ± 8.14 (22∼65)	(A) 3.98 ± 1.02 yr (0.6∼5 yr) (B) 3.94 ± 1.05 yr (0.8∼6 yr)	Modified DXT + (B)	MT: Tuina (1 time/day)	4 weeks	NR	(1) SS (2) OFS (3) RVA-BF (4) LVA-BF (5) BA-BF (6) TER (7) ET level (8) CGRP level	(1) (A) > (B)^*∗*^ (2) (A) > (B)† (3) (A) > (B)^*∗*^ (4) (A) > (B)^*∗*^ (5) (A) > (B)^*∗*^ (6) (A) > (B)^*∗*^ (7) (A) > (B)^*∗*^ (8) (A) > (B)†	NR
Lyu [48]	54 (27 : 27)	China	(A) 35.24 ± 2.15 (20∼59) (B) 31.17 ± 1.53 (18∼60)	NR	Modified BYT + (B)	AT (1 time/day)	20 days	NR	(1) SS (2) RVA-BF (3) LVA-BF (4) BA-BF (5) TER	(1) (A) > (B)^*∗*^ (2) N.S. (3) (A) > (B)^*∗*^ (4) (A) > (B)^*∗*^ (5) (A) > (B)^*∗*^	NR
Pan [49]	100 (50 : 50)	China	(A) 42.41 ± 5.93 (B) 40.87 ± 6.25	(A) 3.91 ± 0.74 mon (B) 4.18 ± 0.81 mon	Modified BBTT + (B)	MT: Tuina (1 time/day)	2 weeks	NR	(1) SS (2) TER (3) ET level (4) CGRP level	(1) (A) > (B)† (2) (A) > (B)^*∗*^ (3) (A) > (B)† (4) (A) > (B)†	Gastrointestinal discomfort (1)
Qin [50]	163 (79 : 84)	China	54.78 ± 10.36	NR	Modified YCT + (B)	AD: betahistine (6 mg·tid)	2 weeks	3 months	(1) SS	(1) (A) > (B)^*∗*^	NR
Qiu [51]	110 (55 : 55)	China	(A) 53.8 ± 5.5 (43∼65) (B) 52.6 ± 4.7 (42∼63)	(A) 4.5 ± 0.7 mon (1∼8 mon) (B) 4.4 ± 0.8 mon (2∼9 mon)	Modified YCT + (B)	AT (1 time/day)	1 month	NR	(1) SS (2) BA-BF (3) TER (4) Fib level (5) TC level	(1) (A) > (B)^*∗*^ (2) (A) > (B)^*∗*^ (3) (A) > (B)^*∗*^ (4) (A) > (B)^*∗*^ (5) (A) > (B)^*∗*^	NR
Shang [52]	82 (41 : 41)	China	40.2 ± 1.7 (31∼67)	3.1 ± 0.5 yr (0.33∼8 yr)	Modified GGT + (B)	MT (qd)	2 weeks	NR	(1) SS (2) TER	(1) (A) > (B)^*∗*^ (2) (A) > (B)^*∗*^	NR
Shang [53]	134 (67 : 67)	China	(A) 36.21 ± 4.74 (19∼63) (B) 36.51 ± 4.43 (18∼64)	(A) 1.35 ± 0.82 yr (2 mon∼5 yr) (B) 1.21 ± 0.78 yr (1 mon∼4 yr)	Modified GGT + (B)	AD: nimodipine (4 mg/day)	2 weeks	NR	(1) RVA-BF (2) LVA-BF (3) BA-BF (4) TER	(1) (A) > (B)† (2) (A) > (B)† (3) (A) > (B)† (4) (A) > (B)^*∗*^	NR
Shen [54]	120 (60 : 60)	China	(A) 54.22 ± 5.31 (42∼67) (B) 54.53 ± 5.07 (43∼66)	NR	Modified YCT + (B)	AT (1 time/day)	NR	NR	(1) SS (2) TER	(1) (A) > (B)† (2) (A) > (B)†	NR
Shi [55]	74 (37 : 37)	China	(A) 54.8 ± 8.9 (B) 55.6 ± 8.4	(A) 3.3 ± 0.9 days (B) 3.5 ± 0.6 days	Modified DXT + (B)	AD: betahistine (12 mg·tid)	2 weeks	NR	(1) RVA-BF (2) LVA-BF (3) BA-BF (4) TER	(1) (A) > (B)^*∗*^ (2) (A) > (B)^*∗*^ (3) (A) > (B)^*∗*^ (4) (A) > (B)^*∗*^	NR
Tan [56]	154 (77 : 77)	China	23.6 ± 2.5 (18∼30)	37.6 ± 7.9 days (7∼60 days)	Modified BBTT + (B)	AD: betahistine (8 mg·bid)	10 days	NR	(1) TER	(1) (A) > (B)†	NR
Wang [57]	66 (34 : 32)	China	(A) 35.34 ± 3.24 (20∼64) (B) 35.63 ± 2.89 (20∼65)	(A) 3.63 ± 1.45 yr (0.2∼10 yr) (B) 3.74 ± 1.63 yr (0.8∼12 yr)	Modified DXT + (B)	MT: Tuina (5 times/week)	4 weeks	NR	(1) SS (2) TER	(1) (A) > (B)† (2) (A) > (B)†	NR
Wang [58]	160 (80 : 80)	China	49.37 ± 7.48 (33∼78)	3.29 ± 1.44 yr (0.5∼9.5 yr)	Modified BBTT + (B)	AD: flunarizine (5 mg·qd)	4 weeks	NR	(1) TER	(1) (A) > (B)^*∗*^	NR
Wang [59]	86 (43 : 43)	China	(A) 44.76 ± 3.69 (23∼67) (B) 45.01 ± 3.12 (22∼68)	(A) 1.04 ± 0.63 yr (4 mon∼2 yr) (B) 1.13 ± 0.64 yr (3 mon∼2 yr)	Modified YCT + (B)	AT (1 time/day)	4 weeks	NR	(1) RVA-BF (2) LVA-BF (3) BA-BF (4) TER (5) Fib level (6) TC level	(1) (A) > (B)† (2) (A) > (B)† (3) (A) > (B)† (4) (A) > (B)^*∗*^ (5) (A) > (B)† (6) (A) > (B)†	NR
Wang [60]	80 (40 : 40)	China	(A) 54.23 ± 9.09 (25∼73) (B) 54.71 ± 9.91 (25∼72)	3.29 ± 1.44 yr (7 days∼3 mon)	Modified BBTT + (B)	AT (5 times/week)	2 weeks	NR	(1) TER	(1) (A) > (B)^*∗*^	Abdominal pain (1) Fainting during acupuncture (1)
Xu [61]	112 (56 : 56)	China	(A) 41.12 ± 3.24 (18∼65) (B) 40.92 ± 3.38 (18∼67)	(A) 2.67 ± 3.24 yr (1∼4 yr) (B) 2.71 ± 0.92 yr (1∼5 yr)	Modified GGT + (B)	MT: Tuina (3 times/day)	4 weeks	NR	(1) SS (2) RVA-BF (3) LVA-BF (4) BA-BF	(1) (A) > (B)^*∗*^ (2) (A) > (B)^*∗*^ (3) (A) > (B)^*∗*^ (4) (A) > (B)^*∗*^	NR
Yang [62]	146 (73 : 73)	China	(A) 35.72 ± 6.66 (18∼54) (B) 35.37 ± 6.51 (19∼55)	(A) 3.14 ± 0.75 mon (1∼5 mon) (B) 3.37 ± 0.81 mon (2∼5 mon)	Modified YCT + (B)	AT (1 time/day)	2 weeks	NR	(1) RVA-BF (2) LVA-BF (3) BA-BF (4) TER	(1) (A) > (B)^*∗*^ (2) (A) > (B)^*∗*^ (3) (A) > (B)^*∗*^ (4) (A) > (B)^*∗*^	NR
Yang [63]	143 (73 : 70)	China	(A) 37.4 ± 1.5 (20∼70) (B) 36.5 ± 1.2 (18∼69)	(A) 2.4 ± 0.3 yr (0.5 mon∼8 yr) (B) 2.5 ± 0.2 yr (1 mon∼7 yr)	Modified DXT + (B)	AD: flunarizine (10 mg·qd) and betahistine (20 mg/day)	2 weeks	6 months	(1) SS (2) RVA-BF (3) LVA-BF (4) BA-BF (5) TER	(1) (A) > (B)^*∗*^ (2) (A) > (B)^*∗*^ (3) (A) > (B)^*∗*^ (4) (A) > (B)^*∗*^ (5) (A) > (B)^*∗*^	Rash (1) Gastrointestinal discomfort (1) Diarrhea (1) Fatigue (2)
Yao [64]	78 (39 : 39)	China	(A) 42.17 ± 4.35 (22∼58) (B) 42.59 ± 5.38 (23∼62)	(A) 5.86 ± 1.35 yr (0.04∼9 yr) (B) 6.19 ± 1.34 yr (0.04∼11 yr)	Modified BBTT + (B)	AT (1 time/5 days)	6 weeks	NR	(1) RVA-BF (2) LVA-BF (3) BA-BF (4) TER	(1) (A) > (B)† (2) (A) > (B)† (3) (A) > (B)† (4) (A) > (B)†	NR
Zhang [65]	290 (145: 145)	China	(A) 57.97 ± 3.54 (47∼76) (B) 58.45 ± 3.36 (46∼76)	(A) 2.56 ± 1.42 yr (1.5∼4.5 yr) (B) 2.85 ± 1.36 yr (1.5∼5 yr)	Modified BBTT + (B)	AT + MT (AT: 1 time/day, MT: Tuina, 1 time/2 days)	4 weeks	NR	(1) OFS (2) TER	(1) (A) > (B)^*∗*^ (2) (A) > (B)^*∗*^	NR
Zhu [66]	120 (60 : 60)	China	(A) NR (31∼59) (B) NR (33∼58)	(A) NR (10 days∼3 yr) (B) NR (7 days∼4 yr)	Modified DXT + (B)	MT: Tuina (1 time/day)	2 weeks	NR	(1) SS (2) RVA-BF (3) LVA-BF (4) BA-BF (5) TER	(1) (A) < (B)† (2) (A) > (B)† (3) (A) > (B)† (4) (A) > (B)^*∗*^ (5) (A) > (B)^*∗*^	NR
Zhu [67]	60 (30 : 30)	China	(A) 45.5 ± 3.4 (20∼67) (B) 42.3 ± 2.1 (21∼65)	(A) NR (5 days∼9 yr) (B) NR (7 days∼10 yr)	Modified GGT + (B)	AT (1 time/day)	2 weeks	NR	(1) SS (2) TER	(1) (A) > (B)^*∗*^ (2) (A) > (B)^*∗*^	NR

Significant differences between the two groups are indicated as follows: ^*∗*^*p* < 0.05 and ^†^*p* < 0.01. Insignificant differences between the two groups (*p* > 0.05) are indicated by N.S. AD, anti-vertigo drug; AE, adverse events; AT, acupuncture therapy; BA-BF, basilar artery blood flow; BBTT, Banxia Baizhu Tianma Tang; BYT, Buzhong Yiqi Tang; CGD, cervicogenic dizziness; CGRP, calcitonin gene-related peptide; DXT, Dingxuan Tang; ET, endothelin; Fib, fibrinogen; GGT, Gegen Tang; GJT, Gegen Jieji Tang; LVA-BF, left vertebral artery blood flow; MT, manual therapy; NR, not reported; OFS, Overall functional score; RVA-BF, right vertebral artery blood flow; SS, simple score; TC, total cholesterol; TER, total effective rate; and YCT, Yiqi Congming Tang.

**Table 2:** Details of the Chinese herbal medicines BBTT, BYT, and DXT in the included studies.

Study ID	Gao [37]	Gu [39]	Huang [42]	Pan [49]	Tan [56]	Wang [58]	Wang [60]	Yao [64]	Zhang [65]	Bai [33]	Lyu [48]	Cheng [35]	Ji [43]	Li [46]	Liu [47]	Shi [55]	Wang [57]	Yang [63]	Zhu [66]
CHM	BBTT									BYT		DXT							
Administration duration and frequency	2 wks, NR	2∼8 wks, tid	1 mon, bid	2 wks, bid	10 dys, bid	4 wks, bid	2 wks, bid	6 wks, bid	4 wks, bid	2 dys, bid	20 dys, bid	2 wks, bid	2 wks, bid	1 mon, bid	4 wks, bid	2 wks, bid	4 wks, bid	2 wks, bid	2 wks, bid
*Atractylodis* Rhizoma Alba	12	7.5	15	10	9	15	12	10	18	15	10		20	10	20	15		10	
Citri Reticulatae Pericarpium	12	6		10			10		12	10	6		10		10	10			
Glycyrrhizae Radix et Rhizoma			9	6	9	9	5	6	3	10	9		5	10	9				10
*Citrus reticulata* Blanco			9		9	9		10											
Gastrodiae Rhizoma	12		12	9	9	12	20	10	15					15	10		12	15	10∼15
Pinelliae Tuber	10	5	10	9	9	10	15	6	12				9	18				10	
Poria Sclerotium	30	7.5	12	10	9	12	30	20	9				15	25	30			10	30
Poria Sclertum Cum Pini Radix																10			
Zingiberis Rhizoma Recens		5	9	10	6	9	9		6					10					
Zizyphi Fructus			3EA	2EA	3EA	3EA	10		9										
*Angelicae* Gigantis Radix										10	10	15	10		15	10			
Bupleuri Radix										10	12				10				
Cimicifugae Rhizoma										10	6								
Codonopsis Pilosulae Radix										10	10		20	25	30	15			
Astragali Radix										30	60	30				20			
*Uncariae* Ramulus Cum Uncus								10									12	15	30
*Salviae miltiorrhizae* Radix																	9	15	15∼30
Polygoni Multiflori Radix																10		10	
Scorpio								3									12	5	
Lumbricus												10				15			
Paeoniae Radix		7.5								10	10	10	10	10	15			10	30
Cinnamomi Ramulus														10	10				
*Puerariae* Radix										20	30	10		20		20	20	20	30∼60
Osterici seu Notopterygii Radix et Ehizoma								10											
Phellodendri Cortex										10	12								
Viticis Fructus	12																		
Cnidii Rhizoma	12							10				10	10	10	15	10	9		15∼30
Alismatis Rhizoma	10								9	20	30		20		20				
Arisaematis Rhizoma	10																		
Magnoliae Cortex	12																		
Phyllostachyos Caulis in Taeniam		7.5														10			
Aurantii Fructus Immaturus		6																	
Myrrha								10											
Olibanum								10											
Fossilia Ossis Mastodi													30		30				
Ostreae Testa													30		30		30		
Nelumbinis Folium															15				
Zingiberis Rhizoma															9				
Margaritiferae Usta Concha																	30		
Loranthi Ramulus et Folium																	12		
Chrysanthmi Flos									10										
Batryticatus Bombyx														6				10	
Notoginseng Radix et Rhizoma												6							
Carthami Flos												10							
Persicae Semen												10							
Aconiti Lateralis Radix Preparata												5							
Rehmanniae Radix Preparata																15			
Cuscutae Semen																15			
Cistanchis Herba																15			
Eucommiae Cortex																15			

BBTT, Banxia Baizhu Tianma Tang; BYT, Buzhong Yiqi Tang; CHM, Chinese herbal medicine; DXT, Dingxuan Tang.

**Table 3:** Details of the Chinese herbal medicines GGT, GJT, and YCT in the included studies.

Study ID	Chen [34]	Huagn [41]	Ju [44]	Shang [52]	Shang [53]	Xu [61]	Zhu [67]	Hu [40]	Dai [36]	Gu [38]	Li [45]	Qin [50]	Qiu [51]	Shen [54]	Wang [59]	Yang [62]
CHM	GGT							GJT	YCT							
Administration duration and frequency	1 mon, bid	2 wks, bid	4 wks, bid	2 wks, bid	2 wks, bid	4 wks, bid	2 wks, bid	2 wks, tid	4 wks, bid	4 wks, tid	4 wks, bid	2 wks, bid	1 mon, bid	NR, bid	4 wks, bid	2 wks, NR
*Atractylodis* Rhizoma Alba									12	20		NR	12	12	20	
Glycyrrhizae Radix et Rhizoma	6	10	6		6	6	10		6	10	6		13	13	10	6
*Citrus reticulata* Blanco										15		NR			15	
Gastrodiae Rhizoma			15	15												
Pinelliae Tuber									12				11	11		
Zingiberis Rhizoma Recens		10	10		6	9	10									
Zizyphi Fructus		15			3EA	9	10									
*Angelicae* Gigantis Radix										15					15	
Bupleuri Radix								NR								
Cimicifugae Rhizoma									9	10	9	NR	7	7	10	9
*Codonopsis pilosulae* Radix										20	15	NR			20	
Ginseng Radix											15		11	11		15
Astragali Radix							15		15	30		NR	12	12	30	15
*Salviae miltiorrhizae* Radix	30								12				14	14		
Polygoni Multiflori Radix									12				11	11		
Scorpio	10							NR								
Lumbricus	15															
Paeoniae Radix	15	20	10	9	12	15	12	NR	15		10		11	11		10
Cinnamomi Ramulus		15	10	9	6	6	12	NR								
*Puerariae* Radix	30	30	20	15	30	30	60	NR	12	15	10	NR	10	10	15	9
Osterici seu Notopterygii Radix et Rhizoma			10					NR								
*Angelicae* Dahuricae Radix				10				NR								
Phellodendri Cortex											3		8	8		3
Viticis Fructus									15	15	15	NR	13	13	15	6
Cnidii Rhizoma	15		10	10	9	9		NR		20		NR			20	
Alismatis Rhizoma										10		NR			10	
Ephedrae Herba		5			9		6									
Ostreae Testa					30											
Polygalae Radix								NR								
Ligustici Tenuissimi Rhizoma et Radix								NR								
Batryticatus Bombyx			6			6										
Notoginseng Radix et Rhizoma							3									
Achyranthis Radix	15															
Chaenomelis Fructus						15										
Lycopodii Herba							15									
Coicis Semen							30									
Lycopi Herba							12									
Eleocharitis Rhizoma												NR				

CHM, Chinese herbal medicine; GGT, Gegen Tang; GJT, Gegen Jieji Tang; NR, not reported; YCT, Yiqi Congming Tang.

**Table 4:** Summary of findings.

Outcomes	No. of participants (RCTs)	Anticipated absolute effects (95% CI)		Relative effect (95% CI)	*I* ^2^ value	Quality of evidence (GRADE)	Comments
		Risk with control group	Risk with CHM group				
*Total analysis*							
OFS	704 (5)	—	SMD 2.31 lower (1.48–3.14 lower)	—	94%	⊕⊕○○ Low	Risk of bias (−1) Inconsistency (−1)
SS	2,289 (22)	—	SMD 1.82 higher (1.26–2.38 higher)	—	97%	⊕○○○ Very low	Risk of bias (−1) Publication bias (−1) Inconsistency (−2)
LVA-BF	1,778 (17)	—	MD 5.70 higher (4.18–7.22 higher)	—	97%	⊕⊕○○ Low	Risk of bias (−1) Inconsistency (−2) Strong association (+1)
RVA-BF	1,778 (17)	—	MD 4.83 higher (3.37–6.29 higher)	—	97%	⊕○○○ Very low	Risk of bias (−1) Inconsistency (−2) Publication bias (−1) Strong association (+1)
BA-BF	1,888 (18)	—	MD 5.58 higher (4.24–6.92 higher)	—	96%	⊕○○○ Very low	Risk of bias (−1) Inconsistency (−2) Publication bias (−1) Strong association (+1)
TER	3,582 (33)	295 per 1,000	450 per 1,000 (419–499)	RR 1.55 (1.42–1.69)	0%	⊕⊕⊕○ Moderate	Risk of bias (−1)
ET level	342 (3)	—	MD 14.57 lower (6.81–22.32 lower)	—	96%	⊕○○○ Very low	Risk of bias (−1) Inconsistency (−2)
CGRP level	426 (4)	—	MD 6.24 higher (4.37–8.11 higher)	—	96%	⊕○○○ Very low	Risk of bias (−1) Inconsistency (−2)
Fib level (vs. AT)	348 (4)	—	MD 0.31 lower (0.12–0.50 lower)	—	97%	⊕○○○ Very low	Risk of bias (−1) Inconsistency (−2)
TC level (vs. AT)	348 (4)	—	MD 0.56 lower (0.31–0.82 lower)	—	71%	⊕⊕○○ Low	Risk of bias (−1) Inconsistency (−1)

*Subgroup analysis according to the comparison types*							
CHM plus AD vs. AD							
OFS (vs. betahistine)	98 (1)	—	MD 7.80 lower (6.02–9.58 lower)	—	N/A	⊕○○○ Very low	Risk of bias (−1) Imprecision (−2)
SS	886 (7)	—	SMD 2.45 higher (1.32–3.58 higher)	—	98%	⊕⊕○○ Low	Risk of bias (−1) Inconsistency (−1)
SS (vs. flunarizine)	264 (3)	—	SMD 2.16 higher (0.44–3.87 higher)	—	97%	⊕⊕○○ Low	Risk of bias (−1) Inconsistency (−1)
SS (vs. betahistine)	363 (2)	—	SMD 1.29 higher (0.34 lower–2.91 higher)	—	98%	⊕○○○ Very low	Risk of bias (−1) Inconsistency (−1) Imprecision (−1)
SS (vs. flunarizine and betahistine)	143 (1)	—	MD 6.98 higher (6.48–7.48 higher)	—	N/A	⊕⊕○○ Low	Risk of bias (−1) Imprecision (−1)
SS (vs. diphenidol)	116 (1)	—	MD 2.67 higher (2.41–2.93 higher)	—	N/A	⊕⊕⊕○ Moderate	Imprecision (−1)
LVA-BF	791 (7)	—	MD 5.39 higher (3.33–7.45 higher)	—	98%	⊕⊕○○ Low	Risk of bias (−1) Inconsistency (−1)
LVA-BF (vs. flunarizine)	226 (2)	—	MD 3.96 higher (1.91–6.01 higher)	—	94%	⊕○○○ Very low	Risk of bias (−1) Inconsistency (−1) Imprecision (−1)
LVA-BF (vs. betahistine)	172 (2)	—	MD 8.73 higher (5.49–11.97 higher)	—	94%	⊕○○○ Very low	Risk of bias (−1) Risk of bias (−1) Inconsistency (−1) Imprecision (−1)
LVA-BF (vs. flunarizine and betahistine)	143 (1)	—	MD 4.59 higher (3.28–5.90 higher)	—	N/A	⊕⊕○○ Low	Risk of bias (−1) Imprecision (−1)
LVA-BF (vs. diphenidol)	116 (1)	—	MD 5.51 higher (4.39–6.63 higher)	—	N/A	⊕⊕⊕○ Moderate	Imprecision (−1)
LVA-BF (vs. nimodipine)	134 (1)	—	MD 2.40 higher (1.90–2.90 higher)	—	N/A	⊕⊕○○ Low	Risk of bias (−1) Imprecision (−1)
RVA-BF	791 (7)	—	MD 5.28 higher (3.38–7.18 higher)	—	97%	⊕⊕○○ Low	Risk of bias (−1) Inconsistency (−1)
RVA-BF (vs. flunarizine)	226 (2)	—	MD 4.80 higher (4.23–5.38 higher)	—	0%	⊕⊕○○ Low	Risk of bias (−1) Imprecision (−1)
RVA-BF (vs. betahistine)	172 (2)	—	MD 7.77 higher (7.17–8.37 higher)	—	25%	⊕⊕○○ Low	Risk of bias (−1) Imprecision (−1)
RVA-BF (vs. flunarizine and betahistine)	143 (1)	—	MD 5.04 higher (3.85–6.23 higher)	—	N/A	⊕⊕○○ Low	Risk of bias (−1) Imprecision (−1)
RVA-BF (vs. diphenidol)	116 (1)	—	MD 4.69 higher (3.77–5.61 higher)	—	N/A	⊕⊕⊕○ Moderate	Imprecision (−1)
RVA-BF (vs. nimodipine)	134 (1)	—	MD 1.82 higher (1.35–2.29 higher)	—	N/A	⊕⊕○○ Low	Risk of bias (−1) Imprecision (−1)
BA-BF	791 (7)	—	MD 5.28 higher (3.97–6.59 higher)	—	92%	⊕⊕○○ Low	Risk of bias (−1) Inconsistency (−1)
BA-BF (vs. flunarizine)	226 (2)	—	MD 4.85 higher (4.04–5.65 higher)	—	0%	⊕⊕○○ Low	Risk of bias (−1) Imprecision (−1)
BA–BF (vs. betahistine)	172 (2)	—	MD 5.70 higher (5.15–6.24 higher)	—	0%	⊕⊕○○ Low	Risk of bias (−1) Imprecision (−1)
BA–BF (vs. flunarizine and betahistine)	143 (1)	—	MD 6.92 higher (5.74–8.10 higher)	—	N/A	⊕⊕○○ Low	Risk of bias (−1) Imprecision (−1)
BA-BF (vs. diphenidol)	116 (1)	—	MD 6.23 higher (4.42–8.04 higher)	—	N/A	⊕⊕⊕○ Moderate	Imprecision (−1)
BA-BF (vs. nimodipine)	134 (1)	—	MD 2.74 higher (2.19–3.29 higher)	—	N/A	⊕⊕○○ Low	Risk of bias (−1) Imprecision (−1)
TER	1,529 (13)	311 per 1,000	461 per 1,000 (420–538)	RR 1.53 (1.35–1.73)	21%	⊕⊕⊕○ Moderate	Risk of bias (−1)
TER (vs. flunarizine)	610 (6)	407 per 1,000	590 per 1,000 (472–773)	RR 1.48 (1.16–1.90)	50%	⊕⊕○○ Low	Risk of bias (−1) Inconsistency (−1)
TER (vs. betahistine)	526 (4)	206 per 1,000	322 per 1,000 (262–459)	RR 1.68 (1.27–2.23)	0%	⊕⊕⊕○ Moderate	Risk of bias (−1)
TER (vs. flunarizine and betahistine)	143 (1)	286 per 1,000	562 per 1,000 (369–858)	RR 1.97 (1.29–3.00)	N/A	⊕⊕○○ Low	Risk of bias (−1) Imprecision (−1)
TER (vs. diphenidol)	116 (1)	259 per 1,000	362 per 1,000 (207–632)	RR 1.40 (0.80–2.44)	N/A	⊕⊕○○ Low	Imprecision (−2)
TER (vs. nimodipine)	134 (1)	328 per 1,000	433 per 1,000 (279–669)	RR 1.32 (0.85–2.04)	N/A	⊕○○○ Very low	Risk of bias (−1) Imprecision (−2)
ET level (vs. diphenidol)	116 (1)	—	MD 11.14 lower (9.49–12.79 lower)	—	N/A	⊕⊕⊕○ Moderate	Imprecision (−1)
CGRP level	200 (2)	—	MD 8.89 higher (0.76 lower–18.54 higher)	—	98%	⊕○○○ Very low	Risk of bias (−1) Inconsistency (−1) Imprecision (−2)
CGRP level (vs. flunarizine)	84 (1)	—	MD 13.89 higher (11.48–16.30 higher)	—	N/A	⊕⊕○○ Low	Imprecision (−2)
CGRP level (vs. diphenidol)	116 (1)	—	MD 4.04 higher (3.68–4.40 higher)	—	N/A	⊕⊕⊕○ Moderate	Imprecision (−1)

*CHM plus MT vs. MT*							
OFS	246 (2)	—	SMD 3.17 lower (6.48 lower–0.15 higher)	—	98%	⊕○○○ Very low	Risk of bias (−1) Inconsistency (−1) Imprecision (−2)
SS	726 (7)	—	SMD 1.33 higher (0.12–2.54 higher)	—	98%	⊕⊕○○ Low	Risk of bias (−1) Inconsistency (−1)
LVA-BF	358 (3)	—	MD 6.24 higher (1.36–11.12 higher)	—	98%	⊕⊕○○ Low	Risk of bias (−1) Inconsistency (−1)
RVA-BF	358 (3)	—	MD 5.62 higher (1.03–10.21 higher)	—	98%	⊕⊕○○ Low	Risk of bias (−1) Inconsistency (−1)
BA-BF	358 (3)	—	MD 4.62 higher (0.32–8.91 higher)	—	97%	⊕⊕○○ Low	Risk of bias (−1) Inconsistency (−1)
TER	614 (6)	235 per 1,000	406 per 1,000 (320–508)	RR 1.71 (1.36–2.16)	0%	⊕⊕⊕○ Moderate	Risk of bias (−1)
ET level	226 (2)	—	MD 16.48 lower (33.31 lower–0.34 higher)	—	98%	⊕○○○ Very low	Risk of bias (−1) Inconsistency (−1) Imprecision (−2)
CGRP level	226 (2)	—	MD 4.63 higher (2.25–7.00 higher)	—	93%	⊕○○○ Very low	Risk of bias (−1) Inconsistency (−1) Imprecision (−1)

*CHM plus AT vs. AT*							
OFS	70 (1)	—	MD 1.91 lower (1.37–2.45 lower)	—	N/A	⊕○○○ Very low	Risk of bias (−1) Imprecision (−2)
SS	677 (8)	—	SMD 1.72 higher (1.33–2.11 higher)	—	79%	⊕⊕○○ Low	Risk of bias (−1) Inconsistency (−1)
LVA-BF	629 (7)	—	MD 5.81 higher (2.92–8.70 higher)	—	95%	⊕⊕○○ Low	Risk of bias (−1) Inconsistency (−1)
RVA-BF	629 (7)	—	MD 4.03 higher (1.05–7.01 higher)	—	96%	⊕⊕○○ Low	Risk of bias (−1) Inconsistency (−1)
BA-BF	739 (8)	—	MD 6.43 higher (2.97–9.89 higher)	—	97%	⊕⊕○○ Low	Risk of bias (−1) Inconsistency (−1)
TER	1,149 (13)	307 per 1,000	471 per 1,000 (405–546)	RR 1.54 (1.32–1.78)	0%	⊕⊕⊕○ Moderate	Risk of bias (−1)

*CHM plus MT plus AT vs. MT plus AT*							
OFS	290 (1)	—	MD 7.06 lower Risk of bias (−1) Imprecision (−1; 6.27–7.85 lower)	—	N/A	⊕⊕⊕○ Moderate	Risk of bias (−1)
TER	290 (1)	290 per 1,000	407 per 1,000 (296–563)	RR 1.40 (1.02–1.94)	N/A	⊕⊕⊕○ Moderate	Risk of bias (−1)

*Subgroup analysis according to the CHM prescription names*							
BBTT plus active controls vs. active controls							
OFS	410 (2)	—	SMD 3.44 lower (0.69–6.20 lower)	—	98%	⊕⊕○○ Low	Risk of bias (−1) Inconsistency (−1)
SS	220 (2)	—	MD 5.15 higher (4.81–5.50 higher)	—	0%	⊕⊕○○ Low	Risk of bias (−1) Imprecision (−1)
LVA-BF	184 (2)	—	MD 4.44 higher (3.18–5.69 higher)	—	71%	⊕○○○ Very low	Risk of bias (−1) Inconsistency (−1) Imprecision (−1)
RVA-BF	184 (2)	—	MD 3.85 higher (2.29–5.41 higher)	—	84%	⊕○○○ Very low	Risk of bias (−1) Inconsistency (−1) Imprecision (−1)
BA-BF	184 (2)	—	MD 3.48 higher (0.04–6.92 higher)	—	95%	⊕○○○ Very low	Risk of bias (−1) Inconsistency (−1) Imprecision (−1)
TER	1,168 (9)	329 per 1,000	486 per 1,000 (424–559)	RR 1.48 (1.29–1.70)	33%	⊕⊕⊕○ Moderate	Risk of bias (−1)
ET level	100 (1)	—	MD 25.13 lower (21.29–28.97 lower)	—	N/A	⊕⊕○○ Low	Risk of bias (−1) Imprecision (−1)
CGRP level	100 (1)	—	MD 5.89 higher (4.78–7.00 higher)	—	N/A	⊕⊕○○ Low	Risk of bias (−1) Imprecision (−1)

*BYT plus active controls vs. active controls*							
SS	134 (2)	—	MD 2.04 higher (1.35–2.72 higher)	—	0%	⊕⊕○○ Low	Risk of bias (−1) Imprecision (−1)
LVA-BF	134 (2)	—	MD 1.72 higher (0.57–2.87 higher)	—	0%	⊕⊕○○ Low	Risk of bias (−1) Imprecision (−1)
RVA-BF	134 (2)	—	MD 1.80 lower (0.72–2.88 lower)	—	0%	⊕⊕○○ Low	Risk of bias (−1) Imprecision (−1)
BA-BF	134 (2)	—	MD 0.43 higher (0.68 lower–1.55 higher)	—	0%	⊕○○○ Very low	Risk of bias (−1) Imprecision (−2)
TER	134 (2)	224 per 1,000	284 per 1,000 (157–511)	RR 1.27 (0.70–2.28)	0%	⊕○○○ Very low	Risk of bias (−1) Imprecision (−2)

*DXT plus active controls vs. active controls*							
OFS	126 (1)	—	MD 5.68 lower (4.36–7.00 lower)	—	N/A	⊕⊕○○ Low	Risk of bias (−1) Imprecision (−1)
SS	715 (7)	—	SMD 1.67 higher (0.20–3.14 higher)	—	98%	⊕⊕⊕○ Moderate	Inconsistency (−1)
LVA-BF	579 (5)	—	MD 5.13 higher (3.87–6.40 higher)	—	78%	⊕⊕○○ Low	Risk of bias (−1) Inconsistency (−1)
RVA-BF	579 (5)	—	MD 5.12 higher (3.42–6.83 higher)	—	90%	⊕⊕○○ Low	Risk of bias (−1) Inconsistency (−1)
BA-BF	579 (5)	—	MD 5.14 higher (2.66–7.62 higher)	—	92%	⊕⊕○○ Low	Risk of bias (−1) Inconsistency (−1)
TER	789 (8)	265 per 1,000	431 per 1,000 (352–517)	RR 1.61 (1.33–1.95)	0%	⊕⊕⊕○ Moderate	Risk of bias (−1)
ET level	242 (2)	—	MD 9.71 lower (6.61–12.81 lower)	—	76%	⊕○○○ Very low	Risk of bias (−1) Inconsistency (−1) Imprecision (−1)
CGRP level	326 (3)	—	MD 6.41 higher (4.15–8.67 higher)	—	97%	⊕⊕○○ Low	Risk of bias (−1) Inconsistency (−1)

*GGT plus active controls vs. active controls*							
OFS	98 (1)	—	MD 7.80 lower (6.02–9.58 lower)	—	N/A	⊕○○○ Very low	Risk of bias (−1) Imprecision (−2)
SS	489 (5)	—	SMD 1.92 higher (0.99–2.85 higher)	—	94%	⊕⊕○○ Low	Risk of bias (−1) Inconsistency (−1)
LVA-BF	579 (5)	—	MD 7.29 higher (3.51–11.07 higher)	—	99%	⊕⊕○○ Low	Risk of bias (−1) Inconsistency (−1)
RVA-BF	579 (5)	—	MD 6.18 higher (3.12–9.24 higher)	—	99%	⊕⊕○○ Low	Risk of bias (−1) Inconsistency (−1)
BA-BF	579 (5)	—	MD 5.19 higher (3.50–6.88 higher)	—	96%	⊕⊕○○ Low	Risk of bias (−1) Inconsistency (−1)
TER	609 (6)	299 per 1,000	485 per 1,000 (395–595)	RR 1.62 (1.32–1.99)	0%	⊕⊕⊕○ Moderate	Risk of bias (−1)

*GJT plus active controls vs. active controls*							
SS	200 (1)	—	MD 2.00 higher (1.75–2.25 higher)	—	N/A	⊕⊕○○ Low	Risk of bias (−1) Imprecision (−1)
TER	200 (1)	88 per 1,000	187 per 1,000 (87–425)	RR 2.19 (0.99–4.86)	N/A	⊕○○○ Very low	Risk of bias (−1) Imprecision (−2)

*YCT plus active controls vs. active controls*							
OFS	70 (1)	—	MD 1.91 lower (1.37–2.45 lower)	—	N/A	⊕○○○ Very low	Risk of bias (−1) Imprecision (−2)
SS	531 (5)	—	SMD 1.79 higher (0.93–2.64 higher)	—	94%	⊕⊕○○ Low	Risk of bias (−1) Inconsistency (−1)
LVA-BF	302 (3)	—	MD 7.63 higher (4.69–10.57 higher)	—	80%	⊕⊕○○ Low	Risk of bias (−1) Inconsistency (−1)
RVA-BF	302 (3)	—	MD 7.34 higher (6.02–8.66 higher)	—	0%	⊕⊕⊕○ Moderate	Risk of bias (−1)
BA-BF	412 (4)	—	MD 11.01 higher (4.46–17.56 higher)	—	96%	⊕⊕○○ Low	Risk of bias (−1) Inconsistency (−1)
TER	682 (7)	328 per 1,000	504 per 1,000 (420–604)	RR 1.54 (1.28–1.84)	0%	⊕⊕⊕○ Moderate	Risk of bias (−1)
Fib level	348 (4)	—	MD 0.31 lower (0.12–0.50 lower)	—	97%	⊕⊕○○ Low	Risk of bias (−1) Inconsistency (−1)
TC level	348 (4)	—	MD 0.56 lower (0.31–0.82 lower)	—	71%	⊕⊕○○ Low	Risk of bias (−1) Inconsistency (−1)

If the evidence of more than 10 studies showed MD <4 for the change in the blood flow velocity in the vertebrobasilar artery or RR >2 for the total effective rate, it was considered that there was a strong association for a treatment effect. AD, anti-vertigo drugs; AT, acupuncture therapy; BA-BF, basal artery blood flow; BBTT, Banxia Baizhu Tianma Tang; BYT, Buzhong Yiqi Tang; CHM, Chinese herbal medicine; CI, confidence interval; CGRP, calcitonin gene-related peptide; DXT, Dingxuan Tang; ET, endothelin; Fib, fibrinogen; GGT, Gegen Tang; GJT, Gegen Jieji Tang; GRADE, the grading of recommendations assessment, development, and evaluation; LVA-BF, left vertebral artery blood flow; MD, mean difference; MT, manual therapy; OFS, overall functional score; RCT, randomised controlled trial; RR, risk ratio; RVA-BF, right vertebral artery blood flow; SMD, standardised mean difference; SS, simple score; TC, total cholesterol; TER, total effective rate; YCT, Yiqi Congming Tang.

**Table 5:** Adjusted quality of evidence derived by sensitivity analysis.

Outcomes	Before SA		After SA		
	Anticipated absoluteeffects (95% CI)	*I* ^2^ value	Anticipated absolute effects (95% CI)	*I* ^2^ value	Adjusted quality of evidence (GRADE)
*Total analysis*					
OFS	SMD 2.31 (1.48–3.14)	94%	SMD 1.81 (1.61–2.00)	49%	⊕⊕⊕○ Moderate
TC level (vs. AT)	MD 0.56 (0.31–0.82)	71%	MD 0.43 (0.27–0.60)	0%	⊕⊕⊕○ Moderate

*Subgroup analysis according to the comparison types*					
CHM plus AD vs. AD					
BA-BF	MD 5.28 (3.97–6.59)	92%	MD 5.65 (5.24–6.06)	48%	⊕⊕⊕○ Moderate

*CHM plus MT vs. MT*					
LVA-BF	MD 6.24 (1.36–11.12)	98%	MD 3.81 (2.84–4.79)	0%	⊕⊕○○ Low
RVA-BF	MD 5.62 (1.03–10.21)	98%	MD 3.48 (2.52–4.44)	0%	⊕⊕○○ Low
BA-BF	MD 4.62 (0.32–8.91)	97%	MD 6.67 (4.73–8.62)	43%	⊕⊕○○ Low

*CHM plus AT vs. AT*					
RVA-BF	MD 4.03 (1.05–7.01)	96%	MD 7.28 (6.33–8.22)	0%	⊕⊕⊕○ Moderate

*Subgroup analysis according to the CHM prescription names*					
DXT plus active controls vs. active controls					
LVA-BF	MD 5.13 (3.87–6.40)	78%	MD 4.56 (3.92–5.20)	48%	⊕⊕○○ Low
RVA-BF	MD 5.12 (3.42–6.83)	90%	MD 4.33 (3.75–4.91)	41%	⊕⊕○○ Low
BA-BF	MD 5.14 (2.66–7.62)	92%	MD 6.45 (5.62–7.28)	0%	⊕⊕○○ Low
CGRP level	MD 6.41 (4.15–8.67)	97%	MD 3.87 (3.57–4.17)	66%	⊕⊕○○ Low

*GGT plus active controls vs. active controls*					
SS	SMD 1.92 (0.99–2.85)	94%	SMD 1.39 (1.16–1.62)	74%	⊕⊕○○ Low
LVA-BF	MD 7.29 (3.51–11.07)	99%	MD 10.33 (9.76–10.90)	0%	⊕⊕⊕○ Moderate
BA–BF	MD 5.19 (3.50–6.88)	96%	MD 5.46 (5.00–5.93)	45%	⊕⊕⊕○ Moderate

*YCT plus active controls vs. active controls*					
SS	SMD 1.79 (0.93–2.64)	94%	SMD 2.13 (1.87–2.38)	0%	⊕⊕⊕○ Moderate
LVA-BF	MD 7.63 (4.69–10.57)	80%	MD 3.47 (3.19–3.75)	0%	⊕⊕○○ Low

AD, anti-vertigo drugs; AT, acupuncture therapy; BA-BF, basal artery blood flow; CHM, Chinese herbal medicine; CI, confidence interval; CGRP, calcitonin gene-related peptide; DXT, Dingxuan Tang; GGT, Gegen Tang; GRADE, the grading of recommendations assessment, development, and evaluation; LVA-BF, left vertebral artery blood flow; MD, mean difference; MT, manual therapy; OFS, overall functional score; RVA-BF, right vertebral artery blood flow; SA, sensitivity analysis; SMD, standardised mean difference; SS, simple score; TC, total cholesterol; YCT, Yiqi Congming Tang.

## Data Availability

All data generated or analysed during this study are included in this article (and its supplementary information files).

## Abbreviations

BBTT: Banxia Baizhu Tianma Tang
BYT: Buzhong Yiqi Tang
CENTRAL: Cochrane central register of controlled trials
CGD: Cervicogenic dizziness
CHM: Chinese herbal medicine
CGRP: Calcitonin gene-related peptide
CI: Confidence interval
DXT: Dingxuan Tang
EMBASE: Excerpta medica database
GGT: Gegen Tang
GJT: Gegen Jieji Tang
MD: Mean difference
MEDLINE: Medical literature analysis and retrieval system online
RCT: Randomised controlled trial
RR: Risk ratio
SA: Sensitivity analysis
SMD: Standardised mean difference
TC: Total cholesterol
YCT: Yiqi Congming Tang.

## Supplementary Materials

Supplementary Materials Supplementary materials are given in the .docx file format. *Appendix A*. Search strategies used in English databases. Description of data: search strategies used in three English databases (MEDLINE, EMBASE, and CENTRAL).
