# Supplementary material for: Chinese Herbal Medicine for Cervicogenic Dizziness: A Systematic Review and Meta-Analysis
Source: Evid Based Complement Alternat Med. 2022 May 9;2022:2425851. doi: 10.1155/2022/2425851 (PMC9110151; doi:10.1155/2022/2425851)
Supplement: Supplementary Materials — are given in the .docx file format. Appendix A. Search strategies used in English databases. Description of data: search strategies used in three English databases (MEDLINE, EMBASE, and CENTRAL). [file 2425851.f1.zip › [CGD] Supplementary material_19Jan22.docx]

**Appendix A. Search strategies used in English databases**

**1. Search strategies used in MEDLINE**

| #1 “Dizziness”[MH] OR “Vertigo”[MH] OR “Syncope”[MH] OR “Linkage Disequilibrium”[MH] OR “Dizziness”[TIAB] OR “Orthostasis”[TIAB] OR “Lightheadedness”[TIAB] OR “Light-Headedness”[TIAB] OR “Light Headedness”[TIAB] OR “Vertigos”[TIAB] OR “Vertigo”[TIAB] OR “Spinning”[TIAB] OR “Syncopes”[TIAB] OR “Fainting”[TIAB] OR “Presyncope”[TIAB] OR “Presyncopes”[TIAB] OR “Syncope”[TIAB] OR “Syncopal”[TIAB] OR “Drop Attack”[TIAB] OR “Drop Attacks”[TIAB] OR “Disequilibrium”[TIAB] OR “Disequilibriums”[TIAB]  #2 “Plants, Medicinal”[MH] OR “Drugs, Chinese Herbal”[MH] OR “Phytotherapy”[MH] OR “Plant Extracts”[MH] OR “Medicinal plants”[TIAB] OR “Medicinal Plant”[TIAB] OR “Plant extracts”[TIAB] OR Herbs[TIAB] OR Herb[TIAB] OR Herbal[TIAB] OR “Phytotherapy”[TIAB]  #3 (Traditional[TIAB] OR Chinese[TIAB]) AND (Medicine[TIAB] OR Drugs[TIAB] OR Drug[TIAB])  #4 “Mahwangbujaseshin-tang”[TIAB] OR “Sopung-san”[TIAB] OR “Dodam-tang”[TIAB] OR “Woohwangchungshim-won”[TIAB] OR “Sahyangsohap-won”[TIAB] OR “Jinmu-tang”[TIAB] OR “Gongjin-dan”[TIAB] OR “Jeungikgwiyong-won”[TIAB] OR “Yeonjueum”[TIAB] OR “Jaeumyeongsin-tang”[TIAB] OR “Hyeongbangjiwhang-tang”[TIAB] OR “Cheonghunhwadam-tang”[TIAB] OR “Woohwangchungshim-won”[TIAB] OR “Yukwooltnag”[TIAB] OR “Yukwool-tang”[TIAB] OR “Daejo-hwan”[TIAB] OR “Cheonmabanhwa-Tang”[TIAB] OR “Banhabaekchulchoenma-tang”[TIAB] OR “Chilgi-tang”[TIAB] OR “Hyeongbangdojok-san”[TIAB] OR “Younggaechulgam-tang”[TIAB] OR “Pyungwi-san”[TIAB] OR “Zizyphi Spinosi Semen”[TIAB] OR “Buzhongyiqi-Tang”[TIAB] OR “Hyeongbangsabaek-san”[TIAB] OR “Dojeokgamgi-tang”[TIAB] OR “Gihwangbaekho-tang”[TIAB] OR “Jaeumkunbi-tang”[TIAB] OR “Daeshiho-tang”[TIAB] OR “Bunsimgi-eum”[TIAB] OR “Hyunggaeyungyo-tang”[TIAB] OR “Gwakhyangjunggi-san”[TIAB] OR “Sunghyangchungi-san”[TIAB] OR “Hyangsayangwi-tang”[TIAB] OR “Palmulgunja-tang”[TIAB] OR “Bosimgunbi-tang”[TIAB] OR “Chengsimyeonja-tang”[TIAB] OR “Hyangsayukgunja-Tang”[TIAB] OR “Goepoong-san”[TIAB] OR “Younggaechulgam-tang”[TIAB] OR “Jaeumgeonbi-tang”[TIAB] OR “Sagunja-tang”[TIAB] OR “Yijin-tang”[TIAB] OR “Leejung-tang”[TIAB] OR “Taeeumjowui-tang”[TIAB] OR “Daekumeumja”[TIAB] OR “yookmijihwang-tang”[TIAB] OR “Bangpungtongsung-san”[TIAB] OR “Yanghyeolgeopung-tang”[TIAB] OR “Samul-tang”[TIAB] OR “Soonkiwhalwheul-tang”[TIAB] OR “Yupoongyangyeong-tang”[TIAB] OR “Gamisachi-tang”[TIAB] OR “Hyangsapyeongwi-san”[TIAB] OR “Yangkyuksanwha-tang”[TIAB] OR “Taeksa-tang”[TIAB] OR “Zexie-tang”[TIAB] OR “Joganiknoe-tang”[TIAB] OR “Samhwangsasim-tang”[TIAB] OR “Yanghyeolgeopung-tang”[TIAB] OR “yangxuequfeng-tang”[TIAB] OR “Yanghyeolgeopung-tang”[TIAB] OR “yangxuequfeng-tang”[TIAB] OR “Choweseuncheng-tang”[TIAB] OR “GamiJihwangyeumja”[TIAB] OR “Sibimijihwang-tang”[TIAB] OR “Mihuedungsikjang-tang”[TIAB] OR “Kamiguibitang”[TIAB] OR “Dodamhwalhultang”[TIAB] OR “Kamiguibi-tang”[TIAB] OR “Dodamhwalhul-tang”[TIAB] OR “Shin-Ki-Hwan”[TIAB] OR “Jengjengamiygin-tang”[TIAB] OR “Arrowroot Puerariae Radix”[TIAB] OR “Salvia miltiorrhiza Bunge”[TIAB] OR “Chrysanthemum indicum L.”[TIAB] OR “Vitex rotundifolia Seeds”[TIAB] OR “Rehmanniae Radix Preparata”[TIAB] OR “Lycii fructus”[TIAB] OR “Disocorea batatas”[TIAB] OR “Injinoryeong-san”[TIAB] OR “Cheonginigeuk-Tang”[TIAB] OR “Melonis Calyx”[TIAB] OR “Yukmijihwangwon”[TIAB] OR “Bosimsahwacheonggan-tang”[TIAB] OR “Hachulbosim-tang”[TIAB] OR “Yukmijihwang-won”[TIAB] OR “Hwaryongibcheoneum”[TIAB] OR “Yeongsindodam-tang”[TIAB] OR “Cheongsinhaeo-tang”[TIAB] OR “Sangcheongbaekbuja-hwan”[TIAB] OR “Junghyeon-tang”[TIAB] OR “Sihogayongmo-tang”[TIAB] OR “Ikgichongmyeong-tang”[TIAB] OR “chongi-tang”[TIAB] OR “Jeongansikpung-tang”[TIAB]  #5 #2 OR #3 OR #4  #6 #1 AND #5 |
| --- |

**2. Search strategies used in EMBASE**

| #1 “Dizziness”/exp OR “Positional dizziness”/exp OR “Vertigo”/exp OR “faintness”/exp OR “Gene linkage Disequilibrium”/exp OR Dizziness:ab,ti OR Orthostasis:ab,ti OR Lightheadedness:ab,ti OR Light-Headedness:ab,ti OR Light Headedness:ab,ti OR Vertigos:ab,ti OR Vertigo:ab,ti OR Spinning:ab,ti OR Syncopes:ab,ti OR Fainting:ab,ti OR Presyncope:ab,ti OR Presyncopes:ab,ti OR Syncope:ab,ti OR Syncopal:ab,ti OR Drop Attack:ab,ti OR Drop Attacks:ab,ti OR Disequilibrium:ab,ti OR Disequilibriums:ab,ti  #2 “Medicinal plant”/exp OR “Phytotherapy”/exp OR “Plant extract”/de OR “Herbaceous agent”/exp OR “Tratidional medicine”/exp OR Medicinal plants:ab,ti OR Medicinal plant:ab,ti OR Plant extracts:ab,ti OR Herbs:ab,ti OR Herb:ab,ti OR Herbal:ab,ti OR Phytotherapy:ab,ti  #3 (Traditional:ab,ti OR Chinese:ab,ti) AND (Medicine:ab,ti OR Drugs:ab,ti OR Drug:ab,ti)  #4 “Mahwangbujaseshin-tang”:ab,ti OR “Sopung-san”:ab,ti OR “Dodam-tang”:ab,ti OR “Woohwangchungshim-won”:ab,ti OR “Sahyangsohap-won”:ab,ti OR “Jinmu-tang”:ab,ti OR “Gongjin-dan”:ab,ti OR “Jeungikgwiyong-won”:ab,ti OR “Yeonjueum”:ab,ti OR “Jaeumyeongsin-tang”:ab,ti OR “Hyeongbangjiwhang-tang”:ab,ti OR “Cheonghunhwadam-tang”:ab,ti OR “Woohwangchungshim-won”:ab,ti OR “Yukwooltnag”:ab,ti OR “Yukwool-tang”:ab,ti OR “Daejo-hwan”:ab,ti OR “Cheonmabanhwa-Tang”:ab,ti OR “Banhabaekchulchoenma-tang”:ab,ti OR “Chilgi-tang”:ab,ti OR “Hyeongbangdojok-san”:ab,ti OR “Younggaechulgam-tang”:ab,ti OR “Pyungwi-san”:ab,ti OR “Zizyphi Spinosi Semen”:ab,ti OR “Buzhongyiqi-Tang”:ab,ti OR “Hyeongbangsabaek-san”:ab,ti OR “Dojeokgamgi-tang”:ab,ti OR “Gihwangbaekho-tang”:ab,ti OR “Jaeumkunbi-tang”:ab,ti OR “Daeshiho-tang”:ab,ti OR “Bunsimgi-eum”:ab,ti OR “Hyunggaeyungyo-tang”:ab,ti OR “Gwakhyangjunggi-san”:ab,ti OR “Sunghyangchungi-san”:ab,ti OR “Hyangsayangwi-tang”:ab,ti OR “Palmulgunja-tang”:ab,ti OR “Bosimgunbi-tang”:ab,ti OR “Chengsimyeonja-tang”:ab,ti OR “Hyangsayukgunja-Tang”:ab,ti OR “Goepoong-san”:ab,ti OR “Younggaechulgam-tang”:ab,ti OR “Jaeumgeonbi-tang”:ab,ti OR “Sagunja-tang”:ab,ti OR “Yijin-tang”:ab,ti OR “Leejung-tang”:ab,ti OR “Taeeumjowui-tang”:ab,ti OR “Daekumeumja”:ab,ti OR “yookmijihwang-tang”:ab,ti OR “Bangpungtongsung-san”:ab,ti OR “Yanghyeolgeopung-tang”:ab,ti OR “Samul-tang”:ab,ti OR “Soonkiwhalwheul-tang”:ab,ti OR “Yupoongyangyeong-tang”:ab,ti OR “Gamisachi-tang”:ab,ti OR “Hyangsapyeongwi-san”:ab,ti OR “Yangkyuksanwha-tang”:ab,ti OR “Taeksa-tang”:ab,ti OR “Zexie-tang”:ab,ti OR “Joganiknoe-tang”:ab,ti OR “Samhwangsasim-tang”:ab,ti OR “Yanghyeolgeopung-tang”:ab,ti OR “yangxuequfeng-tang”:ab,ti OR “Yanghyeolgeopung-tang”:ab,ti OR “yangxuequfeng-tang”:ab,ti OR “Choweseuncheng-tang”:ab,ti OR “GamiJihwangyeumja”:ab,ti OR “Sibimijihwang-tang”:ab,ti OR “Mihuedungsikjang-tang”:ab,ti OR “Kamiguibitang”:ab,ti OR “Dodamhwalhultang”:ab,ti OR “Kamiguibi-tang”:ab,ti OR “Dodamhwalhul-tang”:ab,ti OR “Shin-Ki-Hwan”:ab,ti OR “Jengjengamiygin-tang”:ab,ti OR “Arrowroot Puerariae Radix”:ab,ti OR “Salvia miltiorrhiza Bunge”:ab,ti OR “Chrysanthemum indicum L.”:ab,ti OR “Vitex rotundifolia Seeds”:ab,ti OR “Rehmanniae Radix Preparata”:ab,ti OR “Lycii fructus”:ab,ti OR “Disocorea batatas”:ab,ti OR “Injinoryeong-san”:ab,ti OR “Cheonginigeuk-Tang”:ab,ti OR “Melonis Calyx”:ab,ti OR “Yukmijihwangwon”:ab,ti OR “Bosimsahwacheonggan-tang”:ab,ti OR “Hachulbosim-tang”:ab,ti OR “Yukmijihwang-won”:ab,ti OR “Hwaryongibcheoneum”:ab,ti OR “Yeongsindodam-tang”:ab,ti OR “Cheongsinhaeo-tang”:ab,ti OR “Sangcheongbaekbuja-hwan”:ab,ti OR “Junghyeon-tang”:ab,ti OR “Sihogayongmo-tang”:ab,ti OR “Ikgichongmyeong-tang”:ab,ti OR “chongi-tang”:ab,ti OR “Jeongansikpung-tang”:ab,ti  #5 #2 OR #3 OR #4  #6 #1 AND #5  #7 #6 NOT (“Review”/it OR “Animal experiment”/de OR “In vitro study”/de OR “Nonhuman”/de) |
| --- |

**3. Search strategies used in CENTRAL**

| #1 MeSH descriptor: [Dizziness] explode all trees  #2 MeSH descriptor: [Vertigo] explode all trees  #3 MeSH descriptor: [Syncope] explode all trees  #4 MeSH descriptor: [Linkage Disequilibrium] explode all trees  #5 Dizziness OR Orthostasis OR Lightheadedness OR Light-Headedness OR Light Headedness OR Vertigos OR Vertigo OR Spinning OR Syncopes OR Fainting OR Presyncope OR Presyncopes OR Syncope OR Syncopal OR Drop Attack OR Drop Attacks OR Disequilibrium OR Disequilibriums:ab,ti,kw  #6 #1 OR #2 OR #3 OR #4 OR #5  #7 MeSH descriptor: [Plants, Medicinal] explode all trees  #8 MeSH descriptor: [Phytotherapy] this term only  #9 MeSH descriptor: [Plant Extracts] this term only  #10 MeSH descriptor: [Drugs, Chinese Herbal] explode all trees  #11 Medicinal plants OR Medicinal plant OR Plant extracts OR Herbs OR Herb OR Herbal OR Phytotherapy:ab,ti,kw  #12 (Traditional OR Chinese) AND (Medicinei OR Drugs OR Drug)  #13 “Mahwangbujaseshin-tang” OR “Sopung-san” OR “Dodam-tang” OR “Woohwangchungshim-won” OR “Sahyangsohap-won” OR “Jinmu-tang” OR “Gongjin-dan” OR “Jeungikgwiyong-won” OR “Yeonjueum” OR “Jaeumyeongsin-tang” OR “Hyeongbangjiwhang-tang” OR “Cheonghunhwadam-tang” OR “Woohwangchungshim-won” OR “Yukwooltnag” OR “Yukwool-tang” OR “Daejo-hwan” OR “Cheonmabanhwa-Tang” OR “Banhabaekchulchoenma-tang” OR “Chilgi-tang” OR “Hyeongbangdojok-san” OR “Younggaechulgam-tang” OR “Pyungwi-san” OR “Zizyphi Spinosi Semen” OR “Buzhongyiqi-Tang” OR “Hyeongbangsabaek-san” OR “Dojeokgamgi-tang” OR “Gihwangbaekho-tang” OR “Jaeumkunbi-tang” OR “Daeshiho-tang” OR “Bunsimgi-eum” OR “Hyunggaeyungyo-tang” OR “Gwakhyangjunggi-san” OR “Sunghyangchungi-san” OR “Hyangsayangwi-tang” OR “Palmulgunja-tang” OR “Bosimgunbi-tang” OR “Chengsimyeonja-tang” OR “Hyangsayukgunja-Tang” OR “Goepoong-san” OR “Younggaechulgam-tang” OR “Jaeumgeonbi-tang” OR “Sagunja-tang” OR “Yijin-tang” OR “Leejung-tang” OR “Taeeumjowui-tang” OR “Daekumeumja” OR “yookmijihwang-tang” OR “Bangpungtongsung-san” OR “Yanghyeolgeopung-tang” OR “Samul-tang” OR “Soonkiwhalwheul-tang” OR “Yupoongyangyeong-tang” OR “Gamisachi-tang” OR “Hyangsapyeongwi-san” OR “Yangkyuksanwha-tang” OR “Taeksa-tang” OR “Zexie-tang” OR “Joganiknoe-tang” OR “Samhwangsasim-tang” OR “Yanghyeolgeopung-tang” OR “yangxuequfeng-tang” OR “Yanghyeolgeopung-tang” OR “yangxuequfeng-tang” OR “Choweseuncheng-tang” OR “GamiJihwangyeumja” OR “Sibimijihwang-tang” OR “Mihuedungsikjang-tang” OR “Kamiguibitang” OR “Dodamhwalhultang” OR “Kamiguibi-tang” OR “Dodamhwalhul-tang” OR “Shin-Ki-Hwan” OR “Jengjengamiygin-tang” OR “Arrowroot Puerariae Radix” OR “Salvia miltiorrhiza Bunge” OR “Chrysanthemum indicum L.” OR “Vitex rotundifolia Seeds” OR “Rehmanniae Radix Preparata” OR “Lycii fructus” OR “Disocorea batatas” OR “Injinoryeong-san” OR “Cheonginigeuk-Tang” OR “Melonis Calyx” OR “Yukmijihwangwon” OR “Bosimsahwacheonggan-tang” OR “Hachulbosim-tang” OR “Yukmijihwang-won” OR “Hwaryongibcheoneum” OR “Yeongsindodam-tang” OR “Cheongsinhaeo-tang” OR “Sangcheongbaekbuja-hwan” OR “Junghyeon-tang” OR “Sihogayongmo-tang” OR “Ikgichongmyeong-tang” OR “chongi-tang” OR “Jeongansikpung-tang”  #14 #7 OR #8 OR #9 OR #10 OR #11 OR #12 OR #13  #15 #6 AND #14  #16 #15/trials |
| --- |

Abbreviations: CENTRAL, Cochrane Central Register of Controlled Trials; EMBASE, Excerpta Medica database; MEDLINE, Medical Literature Analysis and Retrieval System Online
